# Multilingual Validation of the First French Version of Munich Dysphagia Test—Parkinson's Disease (MDT-PD) in the Luxembourg Parkinson's Study

**DOI:** 10.3389/fneur.2019.01180

**Published:** 2019-11-11

**Authors:** Janine A. Simons, Michel Vaillant, Geraldine Hipp, Lukas Pavelka, Lara Stute, Claire Pauly, Rejko Krüger

**Affiliations:** ^1^Department of Neurology, University of Lübeck, Lübeck, Germany; ^2^Clinical and Experimental Neuroscience, Luxembourg Centre for Systems Biomedicine, University of Luxembourg, Esch-sur-Alzette, Luxembourg; ^3^Competence Centre for Methodology and Statistics, Luxembourg Institute of Health, Luxembourg, Luxembourg; ^4^Parkinson's Research Clinic, Centre Hospitalier de Luxembourg, Luxembourg, Luxembourg; ^5^Transversal Translational Medicine, Luxembourg Institute of Health (LIH), Strassen, Luxembourg

**Keywords:** parkinsonism, swallowing problems, dysphagia, patient questionnaire, early detection, linguistic and psychometric validation

## Abstract

**Introduction:** The Munich Dysphagia Test for Parkinson's disease (MDT-PD) was initially developed and validated in the German population as a highly sensitive and specific self-reported screening questionnaire to detect early oropharyngeal symptoms and aspiration risk in patients with idiopathic Parkinson's disease (iPD). In order to make this tool accessible for prevention in the French speaking populations worldwide, we performed the first French translation and provide a linguistic and psychometric validation in the unique multilingual environment of the Luxembourg Parkinson's Study.

**Methods:** We performed the translation of the MDT-PD into French according to WHO guidelines and subsequently performed the linguistic validation including native speakers. For psychometric validation, 46 patients with parkinsonism from Luxembourg and the Greater Region without severe cognitive impairment were recruited in the frame of the Luxembourg Parkinson's Study. All patients were fluent in French and German completed the MDT-PD in both languages (three times in total).

**Results:** Linguistic and psychometric validation of the French MDT-PD was reflected by a high test-retest (10/26 questions with K > 0.6 and 10/26 with 0.4 < K ≤ 0.6) and language reliability (12/26 K > 0.6 and 8/26 0.4 < K ≤ 0.6), with an internal consistency for the French (Cronbach's alpha 0.84) and German version (0.87); strong item collinerarity strengthens the internal consistency. No significant differences between MDT-PD score distribution and clinical parameters assessing, for example, disease progression, motor state, or cognition has been observed.

**Conclusion:** Based on a multilingual approach in the Luxembourg Parkinson Study, we validated the translation of the first French MDT-PD as a non-invasive tool for early detection of dysphagia in patients with parkinsonism. The unexpectedly high number of positively screened patients at earlier disease stages indicate options for new prevention strategies in large French speaking populations worldwide. Diagnostic validation using clinical and endoscopic swallowing evaluation will be continued soon.

## Highlights

- First French version of MDT-PD questionnaire provides a non-invasive and easy to administer screening tool for the French speaking population.
- Study was conducted among the patients included in the Luxembourg Parkinson's Study with long term annual follow-up and deep clinical, genetic, and multi-omics phenotypisation that will allow for further analysis and prospective follow-up of the analyzed patients.
- Linguistic and psychometric validation resulted in both high reliability properties and high language validity.
- Highlighting the value of MDT-PD implementation as it allows an early detection of oropharyngeal symptoms as well as aspiration risk and therefore supports early dysphagia treatment, preventing severe clinical complications, and maintaining patients' quality of life.
- The observation of a H&Y stage independent presence of swallowing problems strengthening the hypothesis of dysphagia as an early axial symptom in PD.

## Introduction

Oropharyngeal dysphagia is a frequent symptom in both idiopathic Parkinson's disease (PD) and atypical forms of parkinsonism (aPS). Due to an early loss of oropharyngeal sensibility and an impaired self-awareness a pooled prevalence for PD is reported to be 35% (95% CI 28–41) when simple self-assessment was performed, whereas 82% (95% CI 77–87) when objective instrumental measures were taken into account (1).

Contrary to the generally excepted belief that dysphagia occurs in the advanced stages of PD only, a convincing body of evidence suggests it to be an early symptom with non-negligible consequences (2) such as malnutrition, weight loss, dehydration, or aspiration pneumonia with severe impact on prognosis and quality of life. In order to prevent the clinical complications, a timely diagnostic approach and professional care is essential, thus an accessible non-invasive early screening for dysphagia is required (3, 4).

Besides the currently applied gold standard examination with flexible endoscopic evaluation of swallowing (FEES) or videofluoroscopic swallowing study (VFSS) to diagnose dysphagia, there are up to date only two validated dysphagia questionnaires available specially adapted for PD, the Munich Dysphagia Test-Parkinson's Disease [MDT-PD (4)] and the Swallowing Disturbance questionnaire [SDQ, (5)]. While the SDQ only allows to screen for severe swallowing impairment with aspiration, the MDT-PD was developed among 187 individuals in order to screen for the presence of beginning oropharyngeal symptoms, and to assess the risk for laryngeal aspiration in PD patients (6, 7). The MDT-PD is considered to be a highly sensitive and specific weighted self-reporting outcome questionnaire (8). With respect to the screening results, recommendations and indications for further instrumental diagnostics and appropriate medical therapies can be provided (cf. Supplement 1, Table 1.1 or directly on the MDT-homepage/web application www.mdt-parkinson.com).

The questionnaire contains 26 items, which all have been previously tested for reliability and validity. A short-form description is given in Supplement 1, Table 1.2. It has been cross-nationally translated into other languages including English, however yet there was no French version of the questionnaire available, although 274 million people worldwide from 29 countries use French in their daily life.

The objective of this work was to deliver a first translated version of MDT-PD in French and to determine its validity in the multilingual Luxembourgish population where participants were recruited in the frame of the Luxembourg Parkinson's Study (9). The goal of the study was (i) a linguistic and (ii) a psychometric validation of the French version of MDT-PD questionnaire assessing test-retest and language reliability properties, and construct validity with the focus on patients diagnosed with PD as well as aPS.

## Materials and Methods

### Study Population and Design

The subjects enrolled in this study have been recruited in the scope of the Luxembourg Parkinson's study, a nation-wide, monocentric, descriptive, observational, longitudinal-prospective cohort study with an annual follow-up of patients (9).

All the subjects have signed a written informed consent, and the collection has been approved by the National Ethics Board (CNER Ref: 201407/13) and Data Protection Committee (CNPD Ref: 446/2017).

Additionally, a clinical steering committee composed of different health professionals from Luxembourg involved in PD care has been appointed and supervises the recruitment procedures.

To be included in the study, all subjects must meet either the UK Parkinson's Disease Society Brain Bank Clinical Diagnostic Criteria (10, 11) for PD or the criteria defined for the respective atypical forms of parkinsonism, including progressive supranuclear palsy (PSP) (12, 13), multiple system atrophy (MSA) (12), corticobasal syndrome (CBS) (13), or a secondary form of parkinsonism, vascular parkinsonism (VP) (14), based on internationally established criteria. Furthermore, participants must speak fluently French for the ones implicated in the linguistic validation phase and additionally German, for the group implicated in the psychometric validation phase.

As the MDT is a self-reporting questionnaire, participants with a significant cognitive impairment as defined by a MoCA score < 17 were excluded.

All subjects enrolled in the Luxembourg Parkinson's Study undergo a comprehensive clinical phenotyping including the assessment of motor and non-motor aspects of PD, including (but not limited to) Hoehn & Yahr staging, MDS-UPDRS I–IV, cognitive assessment via MoCA and disease duration since diagnosis. For more details on the study design and protocol cf. Hipp et al. (9).

**Figure 1:**
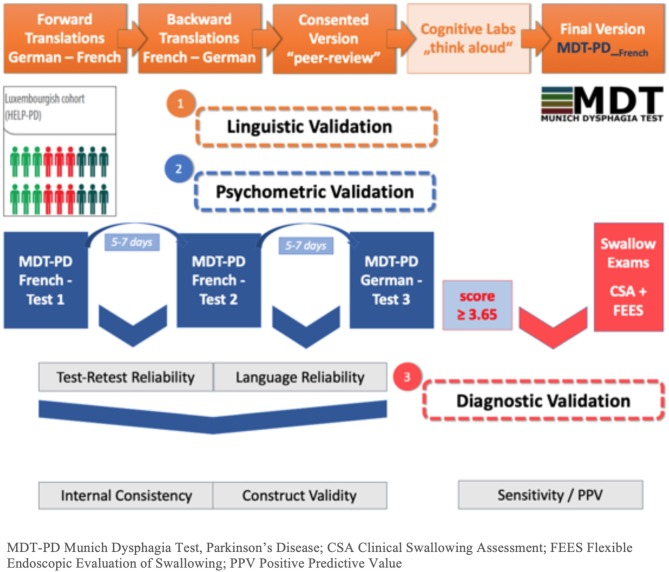
Flowchart of study phases. MDT-PD, Munich Dysphagia Test–Parkinson's Disease; CSA, Clinical Swallowing Assessment; FEES, Flexible Endoscopic Evaluation of Swallowing; PPV, Positive Predictive Value.

The MDT-PD French study has been divided into two phases, (i) the translation and (ii) the validation including (a) linguistic validation of the translation, and (b) the validation of its psychometric aspects, namely test-retest reliability, language reliability, internal consistency, and construct validity. In a continuing third phase the diagnostic validation will be performed separately using the gold standard of clinical and endoscopic swallowing evaluation (cf. Figure 1).

### Questionnaire Description

The questionnaire contains 26 items (Q1–26^1^). Items Q1–Q23 are rated on a 0, 1, 2, and 3 scale, while Q24–26 are rated dichotomously (0, 3). A detailed questionnaire overview is given in Supplement 1.

### Phase I: Translation

In a first step, the German MDT-PD was translated into French with a forward-backward method, by four translators complying with the WHO guidelines for cross-national translations of clinical questionnaires (15).

#### Study Group

The translation group included the copyright holder of the original German test version (speech-language pathologist) and four at least bilingual (fluently German and French speaking at C2 level) experts, one of them with French as mother tongue, including an epidemiologist, a neurologist and two neuropsychologists. Additionally, a group of the Luxembourgish association of speech therapists advised the translation group in terms of technical terminology.

#### Procedure

The ***first step*** involved the forward translation by two translators independently carried out the translation from the original German version into French. Taking the two translations into account, the same translators proceeded to create one consented single version (V1).

The ***second step***, namely the backward translation, consisted in the independent translation into German of the V1, by the two other translators. Here again, both experts proceeded to a joint discussion in order to consent to a single German version (V2).

At the ***final step***, the backward translation V2 was reviewed by the copyright holder and the translators. At this point, both versions V1 and V2 additionally peer-reviewed by a group of the Luxembourgish association of speech therapists.

### Phase II: Validation

#### Linguistic Validation

The preliminary French version, as a result of the translation process, has been tested in a small sample of patients, in order to verify that all the items were interpreted as expected by the population.

##### Subjects

Apart from the general inclusion criteria of the Luxembourg Parkinson's Study and those mentioned above, the patients must additionally speak French as mother tongue. With these inclusion criteria, seven patients have been selected to undergo the questionnaire for the linguistic validation.

##### Procedure

Selected patients were asked to complete the translated version with the “thinking aloud” method. Once they had completed the French preliminary version in front of the investigator, they were asked to rephrase each item in their own words, and to add comments or ask comprehension questions, if necessary. Patients' comments were recorded and analyzed qualitatively.

#### Psychometric Validation

The French version of the MDT-PD questionnaire that has previously been validated linguistically was analyzed for test-retest reliability, language reliability, internal consistency, and construct validity. For more details, cf. Supplement 1, Table 3 that contains the final French MDT-PD version.

##### Subjects

The patients included had to meet the global study inclusion criteria mentioned above, and additionally, as it is the case for most of the Luxembourgish population, they needed to speak fluently French and German. Furthermore, basic medication should be steady during the test completions. Enrollment into the study was performed consecutively.

##### Sample size

Calculations are based on Hong et al. (16), and an evaluation of the agreement between both tests with a Kappa test.

Given the pooled subjectively estimated prevalence of dysphagia of 35% (1) and an expected difference between two proportions of agreement of 0.2 between test and retest, 40 responses to the questionnaire (at the second test) would enable a proportion agreement of 0.65 under H_0_, a proportion agreement of 0.85 under H_1_ and a Kappa value of 0.64 to be detected with a power 0.80 (two-tailed α = 0.05). If the response rate is 100% the minimal number of subjects should be 40.

##### Procedure

The German version of the MDT-PD questionnaire is already included in the yearly visits of the Luxembourg Parkinson's study. The subgroup of this cohort (defined previously in the section Phase II: Validation) were asked to complete the MDT-PD at three different steps (cf. Figure 1). The linguistically validated French MDT-PD questionnaire was sent home before the clinic visit, and patients were asked to fill it out 5–7 days before the visit (FR1). At the clinical visit, the second completion of the French version was performed (FR2). At the date of their clinical visit, the patients were given the German version and asked to fill it out 5–7 days after the visit (GE). The date of completion was carefully recorded on each questionnaire.

The intervals have been chosen in order to avoid that subjects base their answers on the ones given at previous time points (17, 18).

#### General Statistical Methodology

Descriptive statistics were produced as means, standard deviations for the quantitative variables, and as total numbers and percentages for the categorical data, as appropriate to describe the studied population. Chi2 and/or Freeman–Halton test was used to evaluate differences of categorical variables between groups.

Each of the completion versions (FR1, FR2, and GE), were analyzed for internal consistency and construct validity. As for internal consistency, questions were categorized by the amount of change expected and were compared between the test and the retest versions of the questionnaire. Cronbach's alpha was used to compare the French version to the German version. To determine the strength of questionnaire item collinearity, variance inflation factors (VIF) were calculated. Kendall's tau coefficients (t) were calculated to assess bivariate associations of subjects' answers to the MDT-PD items.

Concerning construct validity, to detect inconsistencies between different conditions of PD, patients were categorized in groups depending on diagnosed type of parkinsonism, H&Y staging, disease duration, cognitive state evaluated by the MoCA test, motor performance evaluated by the MDS-UPDRS III and difference in the MDT score compared^2^.

FR1 and FR 2 were compared to analyze test-retest reliability, and FR1 and GE, respectively, FR2 and GE were compared to assess language reliability.

Test-retest and language reliability were estimated for categorical questions using percentage agreement and Kappa or Krippendorff's alpha (KALPHA) values^3^.

Using the FR2 data, exploratory analyses were performed by using interesting questions to create groups of comparison. Patient profiles were then compared. Cohen's Kappa and/or KALPHA (19, 20) was used to evaluate concordance.

## Results

### Results Phase I

Members of the translation group performed modifications based on the feedback of the reviewer and the speech therapists. The modifications concerned technical terms (e.g., *avaler* instead of *déglutir*).

The forward-backward translation method has resulted in a preliminary French version that was agreed on by the four members of the translation group.

### Results Phase II

#### Linguistic Validation

All subjects completed the French MDT-PD successfully. The sample included six patients with IPD and one patient with PSP. Mean age was 66.4 ± 11.5 years, mean H&Y stage was 2.2 ± 0.7, mean MDS-UPDRS III was 35.4 ± 13, and mean MoCA score was 25.1 ± 2.6.

The individual reformulations of the items reflected all the intended meaning. Of the 26 items, three items only (Q3, 19, and 24) were addressed by two patients. The comments concerned scaling of the trouble, but not the interpretation of the item. As this is related to the global structure of the original German version, it was not changed. Consequently, no modification to the initial version was required.

In conclusion, we delivered a linguistically valid French version of the MDT-PD questionnaire in a French population, enabling us to undergo a psychometric validation. Even if the smaller sample size could reduce variability in the feedback, the small amount of comments and the high rate on convergent interpretation by all the subjects gave sufficient arguments that the version provided is linguistically valid.

#### Psychometric Validation

##### Sample description

In total, 46 patients have been recruited from the Luxembourg Parkinson's Study cohort fulfilling the inclusion criteria for validation purpose as mentioned above.

Sex ratio was 37/9 (M/F). The mean MDT was similar at the three test versions (*p* = 0.9354) (cf. Table 1). Additional details of the samples are given in Supplement 2.

**Table 1:** Characteristics of the sample expressed in means for the clinical data and MDT-PD results.

**Variable**	***N***	**Missing data (number of participants)**	**Mean**	**Std. Dev**.	**Min**.	**Max**.
Age (years)	46	0	67.98	9.96	45.47	88.25
H&Y	45	1	2.20	0.76	1.00	5.00
MoCA	45	1	26.04	2.47	18.00	30.00
UPDRS III	43	3	32.51	13.67	7.00	71.00
Disease duration since diagnosis (years)	45	1	7.07	4.37	1.00	20.00
MDT 1 sum score	46	0	3.63	2.35	−0.79	10.47
MDT 2 sum score	39	2	3.75	2.28	−0.76	8.63
MDT 3 sum score	35	3	3.68	2.05	0.16	8.63

*H&Y, Hoehn and Yahr stage; MoCA, Montreal Cognitive Assessment; UPDRS III, Unified Parkinson's Disease Rating Scale part III; MDT 1-3 Munich Dysphagia Test—Parkinson's Disease (1 French version, first test; 2 French version, second test; 3 German version)*.

Thirty-nine patients were diagnosed with IPD, while the remaining patients had a diagnosis of aPS or secondary parkinsonism, namely PSP (*n* = 3), CBS (*n* = 1), and VP (*n* = 1). The two remaining patients had a parkinsonism that had not yet been defined.

##### Response and completion rate

FR1 was completed by all 46 patients, while FR2 was completed by 41 subjects and GE by 38 of the initial group.

In FR2 and GE, the response rate (per test) was close to 100% for a majority of questions except Q22 and Q23 for FR2 and Q10, Q11, Q20, Q23, and Q25 for GE (cf. Supplement 3). Consequently, weighted MDT-PD sum score could not be evaluated in two patients for Fr2 and in three patients for GE due to at least one missing item response (cf. Table 1).

##### Test-retest reliability

In order to analyze test-retest reliability, FR1 responses were compared to FR2 responses.

The number of convergent responses was high in the first test. Up to one-point difference was observed in 20 items and for a high number of patients in item Q12 (*n* = 10), otherwise for less than three patients in the other items (Q3, 4, 8, 15, 20, and 21). Two points divergent responses were found for one to two patients in items Q10, 11, 13, 14, 15, 20, and 22, as well as three points divergent responses in items Q24, 25, and 26 (Note that in items Q24–26 the response possibility was only bivariate (yes/no) with zero or three points. A change in answer behavior at one or two points difference was not possible. In contrast, items Q1–23 had a four-point scale). No item differed in more than three points.

Agreement between FR1 and FR2 was mis-evaluated with Kappa for some questions due to zero cells and more than two categories in the questions. In general, when the Kappa was evaluated, it was in accordance with the KALPHA. There was no dissymmetric evaluation.

Good agreement was found for Q21 for both Kappa (0.82) and KALPHA (0.91). KALPHA was also higher than 0.8 for Q10 (0.84), Q15 (0.80), Q17 (0.84), and Q25 (0.89).

Moderate agreement was found for Q9 (0.77), Q20 (0.74), and Q25 (0.77) with Kappa while only for Q24 (0.66) with KALPHA. Details can be found in Supplement 4, Tables 4.1, 4.2 (item description cf. section Questionnaire Description).

The test-retest reliability in French language confirmed the German language validation as shown by KALPHA (mean – [range] = 0.52 – [0; 0.82] vs. 0.52 – [−0.05; 1]).

##### Language reliability

Concerning FR1 vs. GE, the divergent responses were less important in number of patients concerned and therefore less often compared to the two French versions, except for Q12. Only disagreements with one-point difference (for 1–5 patients) occurred in some cases as well as only four two-point differences for a single patient each (Q11, 13, 14, and 22). Agreement with KALPHA was very good except Q2, Q3, and Q24. Good agreement as measured by the Kappa and KALPHA was showed for Q4 (0.90 resp. 0.83), Q9 (0.84, resp. 0.84), Q10 (0.84 resp. 0.81), and Q25 (1.0 resp. 1.0). KALPHA was also of good agreement for Q16 (0.85) as well as Kappa for Q26 (0.81).

Moderate agreement was concomitantly observed for Q20 (0.79 resp. 0.78), only with Kappa for Q11 (0.74), Q15 (0.73), and Q23 (0.73) while only with KALPHA for Q18 (0.67). Details are provided in Supplement 4, Tables 4.3, 4.4 (item description cf. section Questionnaire Description).

Language reliability was found very good as demonstrated by the KALPHA between the second French test and the original German questionnaire (mean – [range] = 0.69 – [0; 1]).

For the comparison of FR2 with GE, divergent responses occur less often between FR2 vs. GE compared with both French versions. The number of convergent responses was high. Item Q12 was again observed divergent at one difference in points for four patients. Nine items were similarly divergent for one patient, seven items for two patients and one for three patients. A three-points difference was found in item 26 again (0/3 sale) for three patients. The divergences seem lower than previously.

The agreement was generally very high, except for Q2 and Q16. Especially good or excellent agreement was measured by both Kappa and KALPHA (>0.8/1.0) for Q5, Q9, Q11, Q13, Q18, Q19, Q23, and Q26; only by Kappa for Q1, Q6, Q15, Q22, Q2, and only by Kalpha for Q4, Q8, Q10, and Q24.

Concordance for a good agreement was only observed with Kappa for Q6 and Q22 and with KALPHA for Q8 and Q10. For Q1, Q15, and Q25 the agreement was good with Kappa and moderate with KALPHA, while for Q4 it was the opposite.

The agreement was moderate for Q12 for both Kappa and KALPHA, for Q7 with Kappa only, and for Q14 and Q17 for KALPHA. Details are given in Supplement 4, Tables 4.5, 4.6.

**Figure 2:**
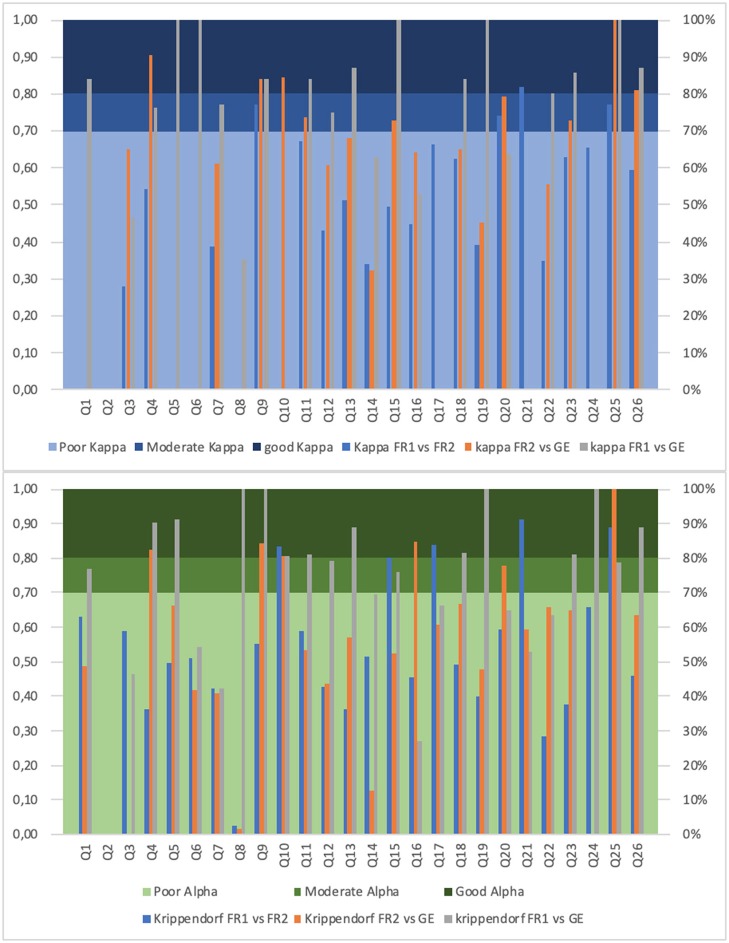
Strength in patients' response agreement for MDT-PD items. The y-axis shows Kappa and Krippendorf Alpha coefficients for the different test-retest and language reliability analyses (including French version 1 and 2, and German version); the x-axis contains the MDT-PD items (Munich Dysphagia Test–Parkinson's Disease, question 1–26). The bar of the histogram is not visible for a KAPPA/KALPHA value of 0.

Figure 2 shows a graphical overview of strength in response agreement of MDT-PD items for all test/language version comparisons, and divided for KAPPA and KALPHA.

##### Internal consistency

The mean relative change between both French questionnaires was below 20%, that can be interpreted as very good, except for items Q12, Q22, and Q26. In general, the change was much lower in comparison of FR2 to GE, while the comparison of the FR1 to GE showed slightly higher changes. Details for each item are given in Supplement 5.

The Cronbach Alpha was evaluated globally and for the deletion of one item^4^. With regard to the achieved internal consistency of the original German study (0.91) (4), the overall values for Cronbach Alpha in the present study was high as well, meaning that the average correlation of items within the test is good (0.80 in FR1, 0.84 in FR2, and 0.83 in GE). While the variances of the items are low, the standardization of the items to a standard deviation of 1 before computing the coefficient alpha did not change it much and maintaining its strength (0.84 for FR1 and 0.87 for GE).

The internal consistency was high both in terms of change between items of the questionnaires and as evaluated by the Cronbach Alpha overall and after deletion of one item (French 1 range = [0.77; 0.82], French 2 range = [0.82; 0.86], German range = [0.80; 0.84]).

Most of the MDT-PD items (22/26) reached a Variance Inflation Factor (VIF) value > 4 for almost one or even for all three language versions demonstrating a strong collinearity with some in particular: Q1 Chewing/swallowing (up to 56.20), Q6 Food gets stuck (40.20), Q4 Multiple swallowing (31.87), Q3 Swallowing trigger (18.81), Q17 Clearing throat (16.90), Q8 Coughing while drinking (15.31), Q15 Off times (14.31), Q20 Rinsing afterwards (18.04), Q19 Tiredness within meals (12.93), Q24 Lung infection (12.34), Q25 Loss of weight (12.04), Q21 Single swallowing (11.84), Q22 Loss of appetite (9.86), Q14 Pills (8.04), and Q10 Changed voice (7.03). Further details are provided in Table 5.4 of the Supplement 5. Bivariate correlation calculation for all MDT-PD items revealed a plurality of moderately positive correlations (t > 0.40). Corresponding correlograms for MDT-PD versions FR1, FR2, and GE are shown in Supplement 5, Figure 5.1.

##### Construct validity

No significant difference between ***diagnoses of parkinsonism*** (including PD, PD with dementia, PSP, CBS, VP, and unspecified parkinsonism) were found for FR1, as the mean MDT total score was ranging from 2 to 5 with an outlier at 7.5 (Cerebrovascular disease with PD features) (*p* = 0.39). Furthermore, we did not find any difference for FR2 (*p* = 0.2) nor for GE (*p* = 0.71) between the different forms of parkinsonism.

Moreover, no significant difference between ***H&Y stages*** were found for FR1, as means were included in the interval 2.5–5 except for an H&Y stage 3 with a higher variability (four patients concerned). The distribution of means was homogeneous with *p* = 0.73. Concerning FR2 and GE, the means were also similarly distributed in the different H&Y stages (*p* = 0.81 and *p* = 0.99, respectively).

No significant relationships of MDT-PD scores were found for the ***MoCA score*** (FR1: R^2^ = 0.017, *p* = 0.39; FR2: R^2^ = 0.021, *p* = 0.38; GE: R^2^ = 0.032, *p* = 0.31), the ***MDS-UPDRS*** ***III score*** (FR1: R^2^ = 0.007, *p* = 0.59; FR2: *R*^2^ = 0.0007, *p* = 0.88; GE: *R*^2^ = 0.014, *p* = 0.51) or ***disease*** ***duration*** (FR1: *R*^2^ = 0.022, *p* = 0.32; FR2: *R*^2^ = 0.0002, *p* = 0.93; GE: *R*^2^ = 0.004, *p* = 0.69).

The categorization of the disease duration in 5 <, 5–10, and >10 y has not led to significant results concerning the effect on the MDT scores at the first, second and third evaluations.

Detailed tables and figures for all clinical parameters assessed are given in Supplement 6.

## Discussion and Conclusion

Based on the results, we were able to create a new language version of the MDT-PD questionnaire in French with very good test-retest and language reliability as well as high internal consistency that can be used as a non-invasive tool for early detection of dysphagia and aspiration risk and will be valuable when implementing novel prevention strategies addressing the French speaking population.

With respect to the currently over 220 million French speakers worldwide, including 72 million so-called partial French speakers (expected to rise to >700 million in 2050 as a result of population growth after estimation of the Organization Internationale de la Francophonie), French is an official language in 29 countries and the sixth most widely spoken language after Mandarin Chinese, English, Hindi, Spanish and Arabic (and the second common language in Europe). At the same time, PD is the second most common age-related neurodegenerative disorder just after Alzheimer's disease with an estimate of approximately seven million people affected worldwide and still exponentially increasing beyond the aging populations with an estimated doubling until 2040 (21). In addition, pneumonia, especially due to dysphagia with aspiration is considered to be the leading cause of mortality in all forms of parkinsonism (22, 23). Considering all of the above mentioned, a screening tool allowing to detect dysphagia in French speaking PD patients becomes indispensable.

Response rate was high at all three MDT-PD tests, and only a few patients dropped out between the tests. The extent of response agreement was globally high, with a majority of up to one-point difference. However, the item Q12 (reflecting a reduced salivation/feeling of dry mouth) showed consistent divergence. This might be due to the variable symptom fluctuation depending on hydration, temperature and momentary diet, which might explain that patients are not consistently responding to this item from one time point to another. For the comparison between the French and German MDT-PD version agreement within questionnaire items was generally higher than for both French versions (especially regarding 2–3 points divergence).

We assume that the chosen interval for MDT-PD completion dates (5–7 days) was optimal and also long enough to exclude that the answers on previous versions can be immediately remembered. In the literature test-retest intervals for PD related instruments/questionnaires usually vary between 2 days up to >1 month without any statistically significant difference, but slightly higher values for 14 days or less (17). Even if we may not suspect a learning effect *per se* in this type of questionnaire (as this is the case in cognitive tests), it might be possible that patients may want to respond consistently from one to another time point. The other way around, a maximum limit of 7 days between two test completions was intended to prevent an answer behavior completely different to the previous test due to changes in the disease condition, e.g., progression of axial symptoms. In addition, there was no modification of medication during the study period.

More generally (and related to Q15), swallowing improvement due to dopaminergic medication is controversially discussed. Although levodopa responsiveness of dysphagia symptoms was formerly considered negligible (24, 25), there are more recent studies showing (short-term) changes in swallowing performance due to oral L-Dopa-intake, subcutaneous apomorphine application, or transdermal rotigotine delivery in some patients (26–29).

In the literature it is presumed that the gastrointestinal system does play a multifaceted role in PD, beginning with the presence of pervasive α-synuclein deposition in the gastrointestinal tract that is involved in the pathogenesis of the disease, and ending with implications of the system affecting several complications including drooling and swallowing problems (30). This implicates the high priority of future studies elucidating the role of the gastrointestinal system on the pathological progression of PD. While it is estimated that the majority of dysphagic PD patients are at progressed disease stage, there are few studies discussing dysphagia as an early symptom, prodromal or even the first sign of PD. Nevertheless, early, severe dysphagia is considered to be more relevant in the Parkinsonian variant of MSA (MSA-P) (31).

For FR1 37% of study patients has been identified as dysphagia positive by the weighted MDT-PD screening questionnaire (41% for FR2 and 46% for GE). Table 2 shows the distribution of dysphagia severity assessed by MDT-PD for all three language versions in patients with PD and aPS (additional tables containing distribution and descriptive statistics of MDT-PD score characteristics is provided in Supplement 7). Although these rates are similar to the relatively low percentage of PD patients subjectively complaining about swallowing problems in comparison to objectively assessed dysphagia via instrumental diagnostic approaches as shown in other studies (1, 32, 33), this is an unexpected high rate with regard to the very mild disease stage in the present study (mean H&Y of 2.2).

**Table 2:** Distribution of dysphagia positive/negative screened patients by MDT-PD, separated for patients with PD and aPD.

**MDT-PD questionnaire**	**MDT-PD score**	**PD**	**Proportion from the participant group (%)**	**aPS + secondary PS**	**Proportion from the participant group (%)**	**All cohort (PD + aPS + secondary PS)**	**Proportion from the cohort (%)**
FR1	*N*	**39**	**100**	**7**	**100**	**46**	**100**
	<3.65	26	67	3	43	29	63
	≥3.65	5	13	2	28.5	7	15
	≥4.79	8	20	2	28.5	10	22
FR2	*N*	**34**	**100**	**5**	**100**	**39**	**100**
	<3.65	21	62	2	40	23	59
	≥3.65	3	9	1	20	4	10
	≥4.79	10	29	2	40	12	31
GE	*N*	**31**	**100**	**4**	**100**	**35**	**100**
	<3.65	18	58	1	25	19	54
	≥3.65	4	13	3	75	7	20
	≥4.79	9	29	0	0	9	26

* <3.65 No noticeable dysphagia; ≥3.65 Noticeable, oropharyngeal dysphagia; ≥4.79 Dysphagia with risk of aspiration; Munich Dysphagia Test—Parkinson's Disease, MDT-PD (FR1/FR2, French version test 1/2; GE, German version); PD, Parkinson's disease; aPS, atypical parkinsonian syndrome; absolute numbers of patients (percentages considering the relative proportions for PD vs. aPS vs. total cohort). Bold values represents total number of values per group*.

Given the fact that there was no significant correlation between the MDT-PD score and characteristics of investigated clinical parameters (disease stage, disease duration, motor functions, cognitive performance or diagnose of parkinsonism), we are confident to pursue the hypothesis that dysphagia could be considered as an early symptom in both PD and aPS.

While using the French version of the MDT questionnaire we were not able to show distinctive frequencies of patients with dysphagia between the different types of parkinsonism or the different levels of motor disease progression tested (Hoehn&Yahr, UPDRSIII, disease duration). According to the analyses carried out so far, it did not discriminate different level of disease or symptoms. It could be a consequence of the small sample size especially in the sub groups, as the majority of participants were idiopathic PD patients. But given the fact that the underlying mechanism of dysphagia progression are still unclear, the present results of the correlation analyses thus assume particular significance. However, the original German questionnaire was designed for the detection of noticeable dysphagia for early stage of PD and an Anova analysis of 38 idiopathic PD patients provided similar results for each variable, where the construct validity was tested (4). Therefore, construct validity should be further explored as the sample used in the current research may not be heterogeneous enough. This is currently tested in our subsequent diagnostic validation study as well as for other language translations/cultural adaptations of the MDT-PD.

Usually, dysphagia is associated with clinical parameters like higher H&Y stage, relevant loss of body weight, drooling/sialorrhea, or dementia (6). The increasing of swallowing impairment with disease stage therefore has been expected, but even significant dysphagia symptoms with penetration or aspiration for at least one consistency has been observed in early PD stages with H&Y of 2 or lower among other recent cohort studies (34). Regarding the association between cognitive impairment and dysphagia we are aware of the fact, that we excluded study subjects with MoCA <17 toward securing deep comprehension performance and assume a positive correlation under real-life conditions.

In general, early screening for dysphagia should be taken into consideration in neurological point of care to enable identification of swallowing impairments including the very early oropharyngeal symptoms and particularly to prevent severe clinical complications or threats to health as well as to quality of life. For sure, there is a limitation for the usage of the MDT-PD questionnaire in daily practice among PD patients with severe dementia or severe depression as these cognitive and neuropsychological states may have a negative influence on the answering behavior and also for the prediction of the test result. The ability of adequately comprehending and answering questionnaire items should be tested therefore prior to the completion of patient-centered, self-reported questionnaires.

In contrast to a recently published clinical study where among others, mismatched parameter classifications were unfortunately applied for the comparison between the three different MDT-PD categories and the dysphagia estimations based on a single diagnostic parameter only (penetration-aspiration scale for the consistency water, PAS H_2_O; more details are provided in Supplement 8) (35), the results of this project, being in line with a simultaneously completed study validating the new Italian version of MDT-PD. These findings underline both the very good psychometric properties of the patient questionnaire and its ability to discriminate between “non-dysphagic” PD patients and dysphagic individuals with “any oropharyngeal symptoms,” or even with “risk of aspiration.” In addition, the strong positive correlation between MDT-PD score and FEES results (incl. PAS H_2_O only) was reconfirmed.

Subsequently, the hypothesis above will be further addressed by the follow-up diagnostic validation of the French MDT-PD version, where all included patients with diagnosed parkinsonism and positively screened by the MDT-PD questionnaire will undergo CSA and FEES (cf. Figure 1, phase 3) complying standardized and Parkinson-specific protocols (4). Following this promising first phases, clinical examinations consisting of CSA and FEES should enable us to verify the diagnostic quality of the French MDT-PD.

## Data Availability Statement

The datasets for this manuscript are not publicly available as they are linked to the Luxembourg Parkinson's Study and its internal regulations. Requests to access the datasets should be directed to MV, mean of contact via email: michel.vaillant@lih.lu.

## Ethics Statement

This study was carried out in accordance with the recommendations of the National Ethics Board (CNER Ref: 201407/13) and Data Protection Committee (CNPD Ref: 446/2017. All subjects gave written informed consent in accordance with the Declaration of Helsinki. The protocol was approved by the National Ethics Board. A clinical steering committee composed of different health professionals from Luxembourg supervises the recruitment procedures.

## Author Contributions

JS: research project (conception, organization, and execution), statistical analyses (design, review, and critique), and manuscript (writing of 1st draft). MV: statistical analyses (design and execution) and manuscript (writing, review, and critique). GH: research project (organization and execution), statistical analyses (review and critique), and manuscript (writing, review, and critique). LP and LS: research project (neurological examinations) and manuscript (writing, review, and critique). CP: research project (neuropsychological examinations). RK: research project (conception and organization) and manuscript (review and critique).

### Conflict of Interest

RK serves as Editorial Board Member of the European Journal of Clinical Investigation, Parkinsonism and Related Disorders and Journal of Neural Transmission. RK has received research grants from Fonds National de Recherche de Luxembourg (FNR; PEARL [FNR/P13/6682797/Krüger] and NCER-PD), the German Research Council (DFG; KR2119/8-1), the Michael J. Fox Foundation, the European Union's Joint Program-Neurodegenerative Diseases (JPND; COURAGE-PD), the European Union's Horizon 2020 research and innovation program (No. 692320), and the Federal Ministry for Education and Research (BMBF; Mito-PD 031 A 430 A), as well as speaker's honoraria and/or travel grants from Abbvie, Zambon, and Medtronic. RK participated as PI or site-PI for industry sponsored clinical trials without receiving honoraria. JS and RK received funding by the Fonds Amélie and Hélène de Fabribeckers, the Fondation Roi Baudouin, Claudie Stein-Lambert as well as by other private donors to execute the presented study. JS also received a postdoctoral fellowship by the University of Luxembourg and works as a visiting researcher at the LCSB. The remaining authors declare that the research was conducted in the absence of any commercial or financial relationships that could be construed as a potential conflict of interest.

## Footnotes

^1^(Q1) I have difficulties with the chewing and swallowing of solid/fibrous/crumbly food (e.g., apples, meat, cracker/chips). (Q2) During meals, food/liquid escapes from the mouth (or the nose). (Q3) I find it difficult to directly/quickly start the swallowing process when taking in liquids or food. (Q4) For the complete swallowing of food/liquids I need to swallow multiple times in a row. (Q5) Food residues remain in my mouth after swallowing. (Q6) During the swallowing process, food gets stuck in my throat/esophagus (maybe I even have to choke). (Q7) During (or after) eating food I have to hawk/cough. (Q8) During (or after) drinking liquids (or eating soup) I have to hawk/cough. (Q9) It happens that I have difficulties breathing/a sense of suffocation when swallowing food or liquids. (Q10) Right after eating food/drinking liquids my voice has changed (e.g., coated/weakened/“wet”/“gargling”). (Q11) I have increased amount of saliva in my mouth/I swallow my saliva too rarely or I have general problems swallowing my saliva/drooling. (Q12) I have a very dry mouth/not enough saliva. (Q13) It happens that I cough or have trouble breathing because I have choked on my saliva/saliva went into my trachea. (Q14) I have problems swallowing pills. (Q15) During the off-phases (off-drug-state/declining levodopa levels) I have more difficulties to swallow. (Q16) I avoid specific foods or textures that often make me choke (e.g., nuts, crumb cake, liquid-filled pralines, and raw vegetable salads). (Q17) It is difficult for me to hawk/cough after I choke in order to clear my throat. (Q18) Nowadays, it takes me more time to eat than it used to (e.g., because I have to chew longer/foods are longer in my mouth due to longer preparation time or more careful swallowing). (Q19) It happens that I get tired during meals (or even fall asleep) and don't finish chewing and swallowing my food. (Q20) During meals I have to have liquids to “flush down” the food in order to be able to better swallow. (Q21) I can only swallow liquids in small lips. (Q22) I have a reduced appetite or pleasure to eat than before (sense of taste and smell are potentially affected). (Q23) I have problems, such as heartburn/frequent burping, sense of lump in the throat/esophagus, sense of pressure behind the breastbone. (Q24) Within the last year I had a lung infection or unclear fever-infections. (Q25) I involuntarily loose body weight. (Q26) I drink <50 oz. of liquid during a given day (equal to suggested minimum of 7–8 glasses/cups water, juice, tea, coffee, and soup).

^2^A Cronbach alpha > 0.8 was considered as good, >0.9 excellent, while alpha <0.7 meant very unreliable results.

^3^Intraclass correlation coefficient was estimated for discrete data. Cohen's Kappa and KALPHA coefficients are reliability measures developed to evaluate the agreement among observers, coders, judges, raters, annotators or measuring devices. While the Kappa applies only to squared tables (2 × 2, 3 × 3, …), KALPHA can be used when there are some unmeasured categories. Good agreement is considered for a Kappa > 0.8 and Alpha > 0.8. Alpha > 0.667 and <0.800 is moderate agreement while below 0.667 agreement is discarded. Kappa <0.7 is considered as bad agreement.

^4^With a maximum of 1, 0.70 has been suggested as an acceptable, >0.8 as good, and >0.9 as excellent reliability coefficient.
