# Supplementary material for: Multilingual Validation of the First French Version of Munich Dysphagia Test—Parkinson's Disease (MDT-PD) in the Luxembourg Parkinson's Study
Source: Front Neurol. 2019 Nov 11;10:1180. doi: 10.3389/fneur.2019.01180 (PMC6859962; doi:10.3389/fneur.2019.01180)
Supplement: Supplementary file 1 [file Presentation_1.zip › Supplementary Files.pdf]

Supplementary Material

Supplement 1: Description of the MDT-PD questionnaire

Table 1.1 MDT-PD score evaluation

The questionnaire result is interpreted following the below scale:

**MDT-PD web application** (www.mdt-parkinson.com)			
**Score**	**Classification**	**Clinical interpretation**	**Further intervention recommendations**
**< 3.65**	No noticeable dysphagia	Based on MDT-PD your patient is not showing any symptomatic dysphagia.	Another screening should be performed within the next 12 months.
**≥ 3.65**	Noticeable, oropharyngeal dysphagia	Based on MDT-PD your patient is showing an abnormality regarding a beginning oropharyngeal dysphagia.	For a differentiated analysis of dysphagia symptoms and decisions on necessary interventions (i.e., professional counseling, dietary adjustments, therapy), the following diagnostic examinations are medically indicated:<br>1. clinical dysphagia-therapeutic assessment (including observation of the intake of a test meal/fluids, at several time points)<br>2. additional, instrumental evaluation of swallowing via endoscopy (FEES) or video-fluoroscopy (VFSS) – preferably during levodopa ON and OFF states
**≥ 4.79**	Dysphagia with risk of aspiration	Based on MDT-PD your patient is facing a high risk of (manifested) dysphagia with laryngeal penetration or aspiration.	1. The patient should be transferred to the hospital department specialized in dysphagia diagnostics including endoscopic (FEES) and/or video-fluoroscopic evaluation of swallowing (VFSS). Assessments during the medication ON and OFF phases are recommended.<br>2. Individually tailored, optimal treatment (medical interventions, dietary adaptation, or dysphagia-therapeutic functional as well as compensatory training) can be started immediately after the analysis of symptoms has been completed.
**Cut-offs for the original study have been computed with a sensitivity of 85% and a specificity of 72% for discrimination between "no noticeable dysphagia" vs. "noticeable, oropharyngeal dysphagia" or with a sensitivity of 90% and specificity of 86% vs. "dysphagia with risk of aspiration", and with similarly high predictive and cross-validated values. Validation study was performed according to a diagnostic set based on 18 parameters using standardized protocols for the gold standard of flexible endoscopic evaluation of swallowing (FEES) and clinical swallowing assessment (CSA), and testing 3 different consistencies (fluid, solid, dry-crumbly) as well as two types of pills/tablets (n=82). For further information, see Simons et al. 2014 or www.mdt-parkinson.com**			

Table 1.2 Short forms of the MDT-PD questionnaire items

Category	Item	Allocated raw values
**I Difficulty swallowing food and liquids**	1. Chewing/swallowing	0,1,2,3
	2. Discharge	0,1,2,3
	3. Swallowing trigger	0,1,2,3
	4. Multiple swallowing	0,1,2,3
	5. Food remains	0,1,2,3
	6. Food gets stuck	0,1,2,3
	7. Coughing while eating	0,1,2,3
	8. Coughing while drinking	0,1,2,3
	9. Problems breathing	0,1,2,3
	10. Changed voice	0,1,2,3
**II Difficulty swallowing independent from food intake**	11. Saliva problems	0,1,2,3
	12. Dry mouth	0,1,2,3
	13. Choking on saliva	0,1,2,3
	14. Pills	0,1,2,3
**III Further swallowing-specific and accompanying burden**	15. Off times	0,1,2,3
	16. Avoidance	0,1,2,3
	17. Clearing throat	0,1,2,3
	18. Duration (for meals)	0,1,2,3
	19. Tiredness (within meals)	0,1,2,3

	20. Rinsing afterwards	0,1,2,3
	21. Single swallowing	0,1,2,3
	22. Loss of appetite	0,1,2,3
	23. Heartburn/lump	0,1,2,3
**IV Swallowing-specific health questions**	24. Lung infection	0,3
	25. Loss of weight	0,3
	26. Fluid intake	0,3

Table 1.3 MDT-PD, French version – Questionnaire content

**Test de Dysphagie de Munich - Maladie de Parkinson**
I **PLAINTES RELATIVES A LA DEGLUTITION survenant en mangeant et en buvant**
Catégories de réponse: 0 (presque) jamais; 1 ponctuellement/chaque mois (une/ plusieurs fois); 2 plus souvent/chaque semaine (une/ plusieurs fois); 3 très souvent/chaque jour (une/ plusieurs fois)
1. J'ai des difficultés pour mâcher et avaler des aliments solides / fibreux / friables. (P.ex. pommes, viande, biscuits/biscottes)<br>2. Pendant les repas les aliments solides / liquides me ressortent de la bouche (ou du nez).<br>3. J'ai du mal à initier le processus de déglutition directement/ rapidement pour les aliments solides et liquides.<br>4. Pour avaler des aliments solides / liquides complétement, je dois déglutir plusieurs fois de suite.<br>5. Après avoir dégluti, j'ai des résidus d'aliments en bouche.<br>6. En avalant les aliments restent coincés dans ma gorge/ mon œsophage. (Éventuellement je régurgite par après)<br>7. En mangeant (ou après), je dois me râcler la gorge / tousser.<br>8. En buvant (ou après) (ou en mangeant une soupe), je dois me racler la gorge/ tousser.<br>9. Il m'arrive d'avoir des difficultés respiratoires / sensations d'étouffement en avalant des aliments solides ou liquides.<br>10 Ma voix à l'air modifiée immédiatement après avoir mangé/ bu. (P.ex. voilée/ affaiblie/ „mouillée“/ „gargouillant“)
II PLAINTES RELATIVES A LA DEGLUTITION indépendamment de la prise alimentaire
Catégories de réponse: 0 ne correspond pas; 1 ne correspond plutôt pas; 2 correspond plutôt; 3 correspond complétement
11. J'ai trop de salive dans ma bouche/ j'avale ma salive trop rarement ou j'ai en général des difficultés à avaler ma salive/ des écoulements de salive de la bouche<br>12. J'ai la bouche très sèche/pas suffisamment de salive.<br>13. Il m'arrive de tousser ou d'avoir des difficultés respiratoires après avoir avalé de travers ma salive/ suite au passage de salive dans la trachée.<br>14. J'ai des difficultés à avaler des comprimés.
III **Autres CONTRAINTES liées à la déglutition**
Catégories de réponse: 0 ne correspond pas; 1 ne correspond plutôt pas; 2 correspond plutôt; 3 correspond complétement

15. Durant les phases "off" (atténuation de l'effet des médicaments L-Dopa), j'ai plus de difficultés pour avaler.

16. J'évite certains aliments ou consistances que j'avale souvent de travers. (P.ex. noix, gâteaux secs, pralines fourrées au liquide, crudités)

17. Libérer mes voies respiratoires en toussant/raclant après avoir avalé de travers me pose des problèmes.

18. Je nécessite plus de temps pour manger qu'autrefois. (P.ex. car je garde les aliments plus longtemps en bouche, je dois mâche plus longtemps/"préparer" ou avaler avec plus de précaution)

19. Il m'arrive de me fatiguer beaucoup durant les repas (ou de m'endormir) et de ne pas finir de mâcher les aliments et les avaler.

20. En mangeant, j'ai besoin de liquide pour "faire passer" les aliments afin de mieux les avaler.

21. Je ne peux boire des liquides que par petites gorgées (isolées).

22. J'ai moins d'appétit et je prends moins de plaisir en mangeant qu'autrefois. (Éventuellement l'odorat/ le goût sont aussi altérés)

23. J'ai des problèmes tels que brûlures d'estomac/ reflux fréquents, sensation de boule dans la gorge/ œsophage ou sensation de pression derrière le sternum.

IV QUESTIONS DE SANTÉ relatives à la déglutition

Catégories de réponse: 0 non, ne correspond pas; 3 oui, correspond

24. Au cours de l'année dernière, j'ai eu une pneumonie ou des infections fébriles d'origine indéterminée.

25. J'ai une perte de poids involontaire.

26. Je bois moins de 1.5 l de liquide par jour. (Correspondant à la quantité minimale recommandée de 7à 8 verres/ tasses d'eau, de jus, de thé, de café ou de soupe)

The MDT-PD is available in several language versions free of charge for clinical implementation: www.mdt-parkinson.com

Each version additionally contains preliminary remarks with patient information, instructions and help for completion.

Supplement 2: Description of the sample

46 participants among the PD patients from the LuxPARK cohort were enrolled in the study out of which 13 at visit 1, 14 at visit 2 and 19 at visit 3.

Table 2.1 Number and percentages of the patients for each visit enrolled in the validation process

Event name	Frequency	Percent	Cumulative	Cumulative
**Visit 1**	13	28.26	13	28.26
**Visit 2**	14	30.43	27	58.70
**Visit 3**	19	41.30	46	100.00

Table 2.2 Characteristics of the sample expressed in means for the different assessments

Variable	N	Missing data (n)	Mean	Std Dev	Minimum	Maximum
**Age** (years)	46	0	67.9808857	9.9617232	45.4700000	88.2482167
**H&Y**	45	1	2.2000000	0.7567875	1.0000000	5.0000000
**MoCA**	45	1	26.0444444	2.4675674	18.0000000	30.0000000
**UPDRS III**	43	3	32.5116279	13.6737010	7.0000000	71.0000000
**Disease duration** since diagnosis (years)	45	1	7.0666667	4.3661924	1.0000000	20.0000000
**MDT 1**	46	0	3.6299783	2.3453462	-0.7890000	10.4700000
**MDT 2**	39	2	3.7544359	2.2793351	-0.7640000	8.6280000
**MDT 3**	35	3	3.6764571	2.0526319	0.1550000	8.6280000

Abbreviations: H&Y: Hoehn and Yahr stage; MoCA: Montreal Cognitive Assessment; UPDRS III: Unified Parkinson's Disease Rating Scale part III, MDT 1-3 Munich Dysphagia Test – Parkinson's Disease (1 French version, first test; 2 French version, second test; 3 German version)

Table 2.3 Gender distribution of the sample

Gender	Frequency	Percent	Cumulative (Frequency)	Cumulative (Percent)
**M**	37	80.43	37	80.43
**F**	9	19.57	46	100.00

Table 2.4 Number of patients for different H&Y stages

Hoehn & Yahr Stage	Frequency	Percent	Cumulative Frequency	Cumulative Percent
**1**	3	6.67	3	6.67
**1,5**	5	11.11	8	17.78
**2**	23	51.11	31	68.89
**2,5**	7	15.56	38	84.44
**3**	4	8.89	42	93.33
**4**	2	4.44	44	97.78
**5**	1*	2.22	45	100.00
***Frequency Missing = 1**				

Table 2.5 Distribution of the diagnosis of parkinsonism forms in the sample at the time of the assessment

Final Diagnosis of Parkinsonism	Frequency	Percent	Cumulative Frequency	Cumulative Percent
**Parkinson's Disease**	38	82.60	38	82.60
**Parkinson's Disease and Dementia**	1	2.17	39	84.78
**Cerebrovascular Disease with Parkinsonism Features**	1	2.17	40	86.95
**Progressive Supranuclear Palsy**	3	6.52	43	93.47
**Cortical-basal Syndrome**	1	2.17	44	95.65
**Parkinsonism Unspecified**	2	4.34	46	100.00

Supplement 3: MDT-PD completion rates

Table 3.1 Completion rates at the different time points (item-wise)

	MDT-PD French Version (FR1)		MDT-PD French Version (FR2)		MDT-PD German Version (GE)	
**Item**	**Cumulative Frequency**	**Cumulative Percent**	**Cumulative Frequency**	**Cumulative Percent**	**Cumulative Frequency**	**Cumulative Percent**
**Q1**	46	100.00	41	100.00	36	94.74
**Q2**	46	100.00	41	100.00	37	97.37
**Q3**	46	100.00	41	100.00	37	97.37
**Q4**	46	100.00	41	100.00	37	97.37
**Q5**	46	100.00	41	100.00	37	97.37
**Q6**	46	100.00	41	100.00	37	97.37
**Q7**	46	100.00	41	100.00	37	97.37
**Q8**	46	100.00	41	100.00	37	97.37
**Q9**	46	100.00	41	100.00	37	97.37
**Q10**	46	100.00	41	100.00	36	94.74
**Q11**	46	100.00	41	100.00	37	97.37
**Q12**	46	100.00	41	100.00	37	97.37
**Q13**	46	100.00	41	100.00	37	97.37
**Q14**	46	100.00	41	100.00	38	100.00
**Q15**	46	100.00	41	100.00	38	100.00
**Q16**	46	100.00	41	100.00	38	100.00
**Q17**	46	100.00	41	100.00	38	100.00

**Q18**	46	100.00	41	100.00	38	100.00
**Q19**	46	100.00	41	100.00	37	97.37
**Q20**	46	100.00	41	100.00	38	100.00
**Q21**	46	100.00	40	97.56	38	100.00
**Q22**	46	100.00	40	97.56	37	97.37
**Q23**	46	100.00	41	100.00	38	100.00
**Q24**	46	100.00	41	100.00	37	97.37
**Q25**	46	100.00	41	100.00	38	100.00
**Q26**	46	100.00	41	100.00	37	97.37

Supplement 4: Details for MDT-PD test-retest reliability

Table 4.1 Disagreement rate for the comparison MDT-PD FR1 vs. FR2 (number of patients, item-wise)

Difference (points)				
**Item**	**0**	**1**	**2**	**3**
**Q1**	35	3	0	0
**Q2**	39	0	0	0
**Q3**	37	2	0	0
**Q4**	38	1	0	0
**Q5**	35	4	0	0
**Q6**	35	4	0	0
**Q7**	34	5	0	0
**Q8**	36	2	0	0
**Q9**	39	0	0	0
**Q10**	35	3	1	0
**Q11**	32	4	2	0
**Q12**	29	10	0	0
**Q13**	32	5	1	0
**Q14**	32	3	1	0
**Q15**	36	1	1	0
**Q16**	34	4	0	0
**Q17**	36	3	0	0

**Q18**	34	4	0	0
**Q19**	35	4	0	0
**Q20**	36	2	1	0
**Q21**	37	2	0	0
**Q22**	30	5	2	0
**Q23**	34	4	0	0
**Q24**	38	1	0	0
**Q25**	37	2	0	0
**Q26**	31	1	0	0

Table 4.2 Strength of response agreement in MDT-PD FR1 vs. FR2 (item-wise, evaluated with Kappa and Krippendorf's Alpha)

Comparison	Disagree	Agree	Kappa	Kappa Lower Limit	Kappa Upper Limit	Krip-pendorf Alpha	K-Alpha Lower Limit	K-Alpha Upper Limit
**Q1**	34	4	0.00	0.00	0.00	*0.63*	0.19	0.85
**Q2**	34	5	0.00	0.00	0.00	*-0.05*	-0.89	0.79
**Q3**	35	4	0.28	-0.23	0.79	*0.59*	-0.09	0.86
**Q4**	34	5	0.54	0.20	0.89	*0.36*	0.13	0.84
**Q5**	33	6	0.00	0.00	0.00	*0.50*	-0.01	0.75
**Q6**	30	9	0.00	0.00	0.00	*0.51*	0.14	0.75
**Q7**	28	11	0.39	0.07	0.71	*0.42*	0.02	0.65

**Q8**	34	4	0.00	0.00	0.00	*0.03*	-0.10	0.88
**Q9**	37	2	0.77	0.47	1.00	*0.55*	0.44	1.00
**Q10**	32	7	0.00	0.00	0.00	*0.84*	0.40	0.89
**Q11**	22	16	0.67	0.48	0.87	*0.59*	0.52	0.89
**Q12**	18	21	0.43	0.21	0.65	*0.43*	0.17	0.61
**Q13**	25	13	0.51	0.27	0.75	*0.36*	0.23	0.73
**Q14**	29	7	0.34	0.03	0.65	*0.52*	-0.10	0.66
**Q15**	32	6	0.50	0.12	0.88	*0.80*	0.11	0.90
**Q16**	33	5	0.45	0.09	0.81	*0.46*	0.02	0.89
**Q17**	30	9	0.66	0.42	0.91	*0.84*	0.36	0.84
**Q18**	23	15	0.62	0.42	0.82	*0.49*	0.33	0.76
**Q19**	32	7	0.39	0.13	0.65	*0.40*	-0.03	0.74
**Q20**	32	7	0.74	0.52	0.97	*0.59*	0.42	0.88
**Q21**	32	7	0.82	0.60	1.00	*0.91*	0.56	1.00
**Q22**	22	15	0.35	0.09	0.60	*0.28*	0.04	0.62
**Q23**	29	9	0.63	0.40	0.86	*0.38*	0.38	0.84
**Q24**	37	2	0.65	0.03	1.00	*0.66*	-0.03	1.00
**Q25**	33	6	0.77	0.47	1.00	*0.89*	0.44	1.00
**Q26**	18	14	0.59	0.35	0.83	*0.46*	0.26	0.80

Table 4.3: Disagreement rate for the comparison MDT-PD FR1 vs.GE (number of patients, item-wise)

Difference (points)				
**Item**	**0**	**1**	**2**	**3**
**Q1**	30	2	0	0
**Q2**	31	1	0	0
**Q3**	31	1	0	0
**Q4**	32	0	0	0
**Q5**	30	2	0	0
**Q6**	31	1	0	0
**Q7**	32	0	0	0
**Q8**	29	2	0	0
**Q9**	31	1	0	0
**Q10**	30	2	0	0
**Q11**	29	1	1	0
**Q12**	27	5	0	0
**Q13**	29	2	1	0
**Q14**	28	1	1	0
**Q15**	31	1	0	0
**Q16**	30	1	0	0

**Q17**	31	1	0	0
**Q18**	27	3	0	0
**Q19**	30	2	0	0
**Q20**	30	2	0	0
**Q21**	28	3	0	0
**Q22**	27	3	1	0
**Q23**	29	2	0	0
**Q24**	32	0	0	0
**Q25**	32	0	0	0
**Q26**	29	0	0	1

Table 4.4 Strength of response agreement in MDT-PD FR1 vs. GE (item-wise, evaluated with Kappa and Krippendorf's Alpha)

Comparison	Disagree	Agree	Kappa	Kappa Lower Limit	Kappa Upper Limit	Krip-pendorf Alpha	K-Alpha Lower Limit	K-Alpha Upper Limit
**Q1**	30	2	0.00	0.00	0.00	*0.49*	0.28	1.00
**Q2**	28	4	-0.05	-0.12	0.03	*-0.04*	-1.00	0.74
**Q3**	31	1	0.65	0.02	1.00	*-0.04*	-0.30	1.00
**Q4**	31	1	0.90	0.72	1.00	*0.83*	0.57	1.00
**Q5**	30	2	0.00	0.00	0.00	*0.66*	0.21	1.00

**Q6**	28	4	0.00	0.00	0.00	*0.42*	0.13	0.90
**Q7**	27	5	0.61	0.28	0.95	*0.41*	0.07	0.83
**Q8**	28	3	0.00	0.00	0.00	*0.02*	-0.11	0.88
**Q9**	31	1	0.84	0.54	1.00	*0.84*	0.53	1.00
**Q10**	29	3	0.84	0.66	1.00	*0.81*	0.68	1.00
**Q11**	19	12	0.74	0.53	0.94	*0.53*	0.44	0.86
**Q12**	20	12	0.61	0.38	0.84	*0.44*	0.36	0.76
**Q13**	23	9	0.68	0.43	0.93	*0.57*	0.30	0.84
**Q14**	25	5	0.32	-0.09	0.74	*0.13*	-0.14	0.74
**Q15**	28	4	0.73	0.36	1.00	*0.52*	0.17	1.00
**Q16**	29	2	0.64	0.19	1.00	*0.85*	-0.06	1.00
**Q17**	29	3	0.00	0.00	0.00	*0.61*	0.35	0.93
**Q18**	20	10	0.65	0.43	0.87	*0.67*	0.37	0.79
**Q19**	27	5	0.45	0.14	0.76	*0.48*	-0.04	0.74
**Q20**	27	5	0.79	0.54	1.00	*0.78*	0.56	1.00
**Q21**	25	6	0.00	0.00	0.00	*0.59*	0.19	0.90
**Q22**	22	9	0.55	0.27	0.83	*0.66*	0.22	0.71
**Q23**	25	6	0.73	0.49	0.97	*0.65*	0.42	0.94
**Q24**	32	0	0.00	0.00	0.00	*0.00*	-1.00	0.00
**Q25**	29	3	1.00	1.00	1.00	*1.00*	0.63	1.00
**Q26**	16	14	0.81	0.61	1.00	*0.63*	0.53	0.90

Table 4.5 Disagreement rate for the comparison MDT-PD FR2 vs. GE (number of patients, item-wise)

Difference (points)				
**Item**	**0**	**1**	**2**	**3**
**Q1**	31	1	0	0
**Q2**	31	1	0	0
**Q3**	31	1	0	0
**Q4**	30	2	0	0
**Q5**	32	0	0	0
**Q6**	32	0	0	0
**Q7**	32	0	0	0
**Q8**	30	2	0	0
**Q9**	31	1	0	0
**Q10**	30	2	0	0
**Q11**	30	1	0	0
**Q12**	28	4	0	0
**Q13**	31	1	0	0
**Q14**	31	1	0	0
**Q15**	32	0	0	0
**Q16**	31	1	0	0
**Q17**	30	2	0	0
**Q18**	29	2	0	0

**Q19**	32	0	0	0
**Q20**	29	2	0	0
**Q21**	28	3	0	0
**Q22**	29	2	0	0
**Q23**	31	1	0	0
**Q24**	32	0	0	0
**Q25**	32	0	0	0
**Q26**	30	0	0	2

Table 4.6 Strength of response agreement in MDT-PD FR2 vs. GE (item-wise, evaluated with Kappa and Krippendorf's Alpha)

Comparison	Disagree	Agree	Kappa	Kappa Lower Limit	Kappa Upper Limit	Krip-pendorf Alpha	K-Alpha Lower Limit	K-Alpha Upper Limit
**Q1**	31	1	0.84	0.54	1.00	*0.77*	0.43	1.00
**Q2**	31	1	0.00	0.00	0.00	*-1.00*	-1.00	0.00
**Q3**	30	2	0.47	-0.16	1.00	*0.46*	0.10	1.00
**Q4**	30	2	0.76	0.46	1.00	*0.90*	0.51	1.00
**Q5**	31	1	1.00	1.00	1.00	*0.91*	0.73	1.00
**Q6**	32	0	1.00	1.00	1.00	*0.54*	0.43	1.00
**Q7**	29	3	0.77	0.47	1.00	*0.42*	0.23	0.90
**Q8**	29	3	0.35	-0.22	0.92	*1.00*	-0.09	1.00

**Q9**	31	1	0.84	0.54	1.00	*1.00*	0.53	1.00
**Q10**	28	4	0.00	0.00	0.00	*0.81*	0.55	1.00
**Q11**	20	11	0.84	0.67	1.00	*0.81*	0.57	0.95
**Q12**	22	10	0.75	0.55	0.95	*0.79*	0.54	0.92
**Q13**	25	7	0.87	0.70	1.00	*0.89*	0.61	1.00
**Q14**	27	5	0.63	0.24	1.00	*0.70*	0.29	1.00
**Q15**	30	2	1.00	1.00	1.00	*0.76*	0.64	1.00
**Q16**	28	4	0.53	0.06	1.00	*0.27*	-0.22	0.88
**Q17**	27	5	0.00	0.00	0.00	*0.66*	0.39	0.93
**Q18**	23	8	0.84	0.68	1.00	*0.82*	0.63	0.95
**Q19**	30	2	1.00	1.00	1.00	*1.00*	0.70	1.00
**Q20**	25	6	0.64	0.33	0.94	*0.65*	0.29	0.93
**Q21**	25	6	0.00	0.00	0.00	*0.53*	0.15	0.81
**Q22**	23	8	0.80	0.58	1.00	*0.64*	0.53	0.95
**Q23**	27	5	0.86	0.67	1.00	*0.81*	0.49	0.94
**Q24**	32	0	0.00	0.00	0.00	*1.00*	1.00	1.00
**Q25**	29	3	1.00	1.00	1.00	*0.79*	0.68	1.00
**Q26**	18	14	0.87	0.70	1.00	*0.89*	0.67	1.00

Supplement 5: MDT-PD - Internal consistency

Table 5.1 MDT-PD language comparisons (mean differences item-wise)

	FR2-FR1					GE-FR2					GE-FR1				
**Item**	**N**	**Mean**	**Std Dev**	**Min.**	**Max.**	**N**	**Mean**	**Std Dev**	**Min.**	**Max.**	**N**	**Mean**	**Std Dev**	**Min.**	**Max.**
**Q1**	41	0.09	0.25	0	1	36	0.04	0.18	0	1	37	0.03	0.11	0	0.5
**Q2**	41	0.06	0.17	0	0.5	36	0.03	0.17	0	1	37	0.07	0.21	0	1
**Q3**	41	0.07	0.24	0	1	36	0.04	0.18	0	1	37	0.05	0.23	0	1
**Q4**	41	0.09	0.22	0	1	36	0.06	0.23	0	1	37	0.04	0.18	0	1
**Q5**	41	0.12	0.29	0	1	36	0.01	0.08	0	0.5	37	0.07	0.24	0	1
**Q6**	41	0.13	0.27	0	1	36	0.03	0.12	0	0.5	37	0.06	0.16	0	0.5
**Q7**	41	0.20	0.35	0	1	36	0.06	0.16	0	0.5	37	0.08	0.19	0	0.5
**Q8**	41	0.09	0.26	0	1	36	0.07	0.24	0	1	37	0.10	0.27	0	1
**Q9**	41	0.02	0.11	0	0.5	36	0.03	0.17	0	1	37	0.03	0.16	0	1
**Q10**	41	0.11	0.28	0	1	36	0.05	0.19	0	1	36	0.02	0.10	0	0.5
**Q11**	41	0.18	0.47	0	2	35	0.07	0.18	0	0.67	36	0.14	0.38	0	2
**Q12**	41	0.28	0.44	0	2	36	0.10	0.26	0	1	37	0.20	0.41	0	2
**Q13**	41	0.18	0.40	0	2	36	0.06	0.20	0	1	37	0.16	0.39	0	2
**Q14**	41	0.19	0.41	0	2	36	0.06	0.20	0	1	37	0.17	0.40	0	2
**Q15**	41	0.11	0.37	0	2	37	0.02	0.11	0	0.67	38	0.06	0.21	0	1
**Q16**	41	0.10	0.29	0	1	37	0.09	0.26	0	1	38	0.07	0.25	0	1
**Q17**	41	0.12	0.29	0	1	37	0.05	0.14	0	0.5	38	0.06	0.15	0	0.5

**Q18**	41	0.13	0.22	0	0.67	37	0.05	0.15	0	0.67	38	0.13	0.23	0	0.67
**Q19**	41	0.11	0.26	0	1	37	0.03	0.16	0	1	38	0.11	0.27	0	1
**Q20**	41	0.11	0.36	0	2	36	0.08	0.23	0	1	37	0.05	0.20	0	1
**Q21**	41	0.04	0.17	0	1	37	0.09	0.26	0	1	38	0.08	0.25	0	1
**Q22**	40	0.31	0.56	0	2	36	0.12	0.30	0	1	38	0.26	0.52	0	2
**Q23**	40	0.11	0.26	0	1	36	0.07	0.21	0	1	37	0.08	0.22	0	1
**Q24**	41	0.07	0.47	0	3	37	0.00	0.00	0	0	38	0.08	0.49	0	3
**Q25**	41	0.15	0.65	0	3	37	0.02	0.12	0	0.75	37	0.08	0.49	0	3
**Q26**	41	0.27	0.69	0	3	37	0.24	0.83	0	3	38	0.22	0.70	0	3

Table 5.2 Cronbach's alpha values for MDT-PD language versions

**FR1**		**FR2**		**GE**	
**Raw**	**Standardized**	**Raw**	**Standardized**	**Raw**	**Standardized**
0.80	0.84	0.84		0.83	0.87

Explanation: If the variances of the items vary widely standardized values are obtained by scaling the variables to a unit variance of 1.

Table 5.3 Cronbach's alpha values for MDT-PD language versions (1 item deleted)

The deletion of one item had no major influence on the coefficient Alpha thereby keeping a high level of consistency.

	FR1				FR2				GE			
	Raw		Standardized		Raw		Standardized		Raw		Standardized	
Del. Var.	Corr. with Total	Alpha	Corr. with Total	Alpha	Corr. with Total	Alpha	Corr. with Total	Alpha	Corr. with Total	Alpha	Corr. with Total	Alpha
**Q1**	0.30	0.79	0.37	0.84	0.59	0.82	0.66	0.87	0.38	0.82	0.52	0.86
**Q2**	0.09	0.80	0.13	0.85	-	-	-	-	0.00	0.83	-0.04	0.87
**Q3**	0.51	0.79	0.57	0.83	0.43.	0.83	0.51	0.88	0.50	0.82	0.58	0.86
**Q4**	0.41	0.79	0.46	0.84	0.31.	0.83	0.40	0.88	0.45	0.82	0.53	0.86
**Q5**	0.30	0.79	0.35	0.84	0.33	0.83	0.36	0.88	0.33	0.82	0.34	0.86
**Q6**	0.36	0.79	0.45	0.84	0.59	0.82	0.67	0.87	0.41	0.82	0.51	0.86
**Q7**	0.47	0.78	0.55	0.83	0.58.	0.82	0.60	0.87	0.51	0.82	0.57	0.86
**Q8**	0.29	0.79	0.26	0.84	0.51	0.8	0.55	0.87	0.30	0.82	0.42	0.86
**Q9**	0.55	0.79	0.59	0.83	0.48	0.83	0.47	0.88	0.54	0.82	0.55	0.86
**Q10**	0.42	0.79	0.43	0.84	0.47	0.82	0.48	0.88	0.43	0.82	0.47	0.86
**Q11**	0.47	0.78	0.45	0.84	0.48.	0.82	0.47	0.88	0.47	0.82	0..44	0.86
**Q12**	0.10	0.80	0.09	0.85	0.2.	0.83	0.31	0.88	0.19	0.83	0.21	0.87
**Q13**	0.54	0.78	0.57	0.83	0.62	0.82	0.61	0.87	0.59	0.81	0.56	0.86
**Q14**	0.37	0.79	0.39	0.84	0.28	0.83	0.27	0.88	0.30	0.82	0.27	0.87
**Q15**	0.65	0.77	0.63	0.83	0.62	0.82	0.64	0.87	0.70	0.81	0.72	0.85
**Q16**	0.57	0.78	0.58	0.83	0.63	0.82	0.64	0.87	0.58	0.82	0.58	0.86

**Q17**	0.65	0.77	0.65	0.83	0.69	0.81	0.70	0.87	0.72	0.81	0.75	0.85
**Q18**	0.50	0.78	0.50	0.83	0.28	0.83	0.28	0.88	0.53	0.81	0.51	0.86
**Q19**	0.11	0.80	0.10	0.85	0.29	0.83	0.28	0.88	0.21	0.82	0.20	0.87
**Q20**	0.63	0.78	0.67	0.83	0.65	0.82	0.65	0.87	0.63	0.81	0.69	0.85
**Q21**	0.40	0.79	0.41	0.84	0.47	0.82	0.48	0.88	0.64	0.81	0.67	0.85
**Q22**	0.46	0..78	0..42	0.84	0.57	0.82	0.58	0.87	0.54	0.81	0.51	0.86
**Q23**	0.05	0..80	0..03	0.85	0.21	0.83	0.15	0.89	0.10	0.83	0.01	0.87
**Q24**	0.15	0.80	0..19	0.84	0.31	0.83	0.28	0.88	0.11	0.83	0..05	0.87
**Q25**	0.11	0.80	0..04	0.85	0.26	0.84	0.29	0.88	0.17	0.83	0.11	0.87
**Q26**	0.10	0.82	0.09	0.85	0.01	0.86	-0.03	0.89	017	0.85	0.16	0.87

Table 5.4 Variance Inflation Factor (VIF) values for each question of the MDT-PD questionnaire

		**Variance Inflation**		
**Variable**	**Label** (questionnaire item)	**FR1**	**FR2**	**GE**
**mdt_1**	I have difficulties with the chewing and swallowing of solid/ fibrous/ crumbly food. (e.g. apples, meat, cracker/ chips)	6.12	33.50	56.20
**mdt_2**	During meals, food/liquid escapes from the mouth (or the nose).	2.67		2.83
**mdt_3**	I find it difficult to directly/ quickly start the swallowing process when taking in liquids or food.	6.41	10.58	18.81
**mdt_4**	For the complete swallowing of food/ liquids I need to swallow multiple times in a row.	3.39	16.76	31.87
**mdt_5**	Food residues remain in my mouth after swallowing.	2.69	3.10	4.87
**mdt_6**	During the swallowing process, food gets stuck in my throat/ esophagus. (maybe I even have to choke)	7.68	40.20	28.40
**mdt_7**	During (or after) eating food I have to hawk/cough.	4.01	4.17	5.20
**mdt_8**	During (or after) drinking liquids (or eating soup) I have to hawk/ cough.	2.50	15.31	4.62
**mdt_9**	It happens that I have difficulties breathing/a sense of suffocation when swallowing food or liquids.	4.18	5.26	4.13
**mdt_10**	Right after eating food/ drinking liquids my voice has changed. (e.g. coated/weakened/wet/gargling)	3.94	4.08	7.03

**mdt_11**	I have increased amount of saliva in my mouth /I swallow my saliva too rarely or I have general problems swallowing my saliva/ drooling.	3.03	2.65	3.49
**mdt_12**	I have a very dry mouth/ not enough saliva.	2.95	2.53	3.93
**mdt_13**	It happens that I cough or have trouble breathing because I have choked on my saliva/saliva went into my trachea.	5.30	6.22	5.00
**mdt_14**	I have problems swallowing pills.	2.22	7.01	8.09
**mdt_15**	During the off-phases (off-drug-state/declining levodopa levels) I have more difficulties to swallow.	3.73	7.10	14.31
**mdt_16**	I avoid specific foods or textures that often make me choke. (e.g. nuts, crumb cake, liquid-filled pralines, raw vegetable salads)	4.32	6.36	4.49
**mdt_17**	It is difficult for me to hawk/ cough after I choke in order to clear my throat.	3.83	12.68	16.90
**mdt_18**	Nowadays, it takes me more time to eat than it used to. (e.g. because I have to chew longer/foods are longer in my mouth due to longer preparation time or more careful swallowing)	3.64	3.65	4.62
**mdt_19**	It happens that I get tired during meals (or even fall asleep) and don't finish chewing and swallowing my food.	2.88	6.20	12.93
**mdt_20**	During meals I have to have liquids to flush down the food in order to be able to better swallow.	5.48	11.40	18.04
**mdt_21**	I can only swallow liquids in small sips.	2.78	5.33	11.84
**mdt_22**	I have a reduced appetite or pleasure to eat than before. (sense of taste and smell are potentially affected)	3.26	9.86	8.17
**mdt_23**	I have problems, such as heartburn/ frequent burping, sense of lump in the throat/ esophagus, sense of pressure behind the breastbone.	2.75	3.64	5.86
**mdt_24**	Within the last year I had a lung infection or unclear fever-infections	3.71	12.34	5.21
**mdt_25**	I involuntarily loose body weight.	4.19	8.14	12.04
**mdt_26**	I drink less than 50 oz. of liquid during a given day (equal to suggested minimum of 7-8 glasses/cups water, juice, tea, coffee, soup)	2.07	2.36	3.01

Figure 5.1 Correlograms of the MDT-PD items (FR1, FR2 and GE)

Kendall's tau coefficients (t) show the strength of item correlation (associations of subjects' answers to the MDT-PD items).

Conservative interpretation of (positive or negative) coefficient values (Fowler, Cohen et al. 2006):
0.00–0.19 very week correlation; 0.20–0.39 weak correlation; 0.40–0.69 modest correlation; 0.70–0.89 strong correlation; 0.90–1.00 very strong correlation

a) French version 1 (FR1), n=46

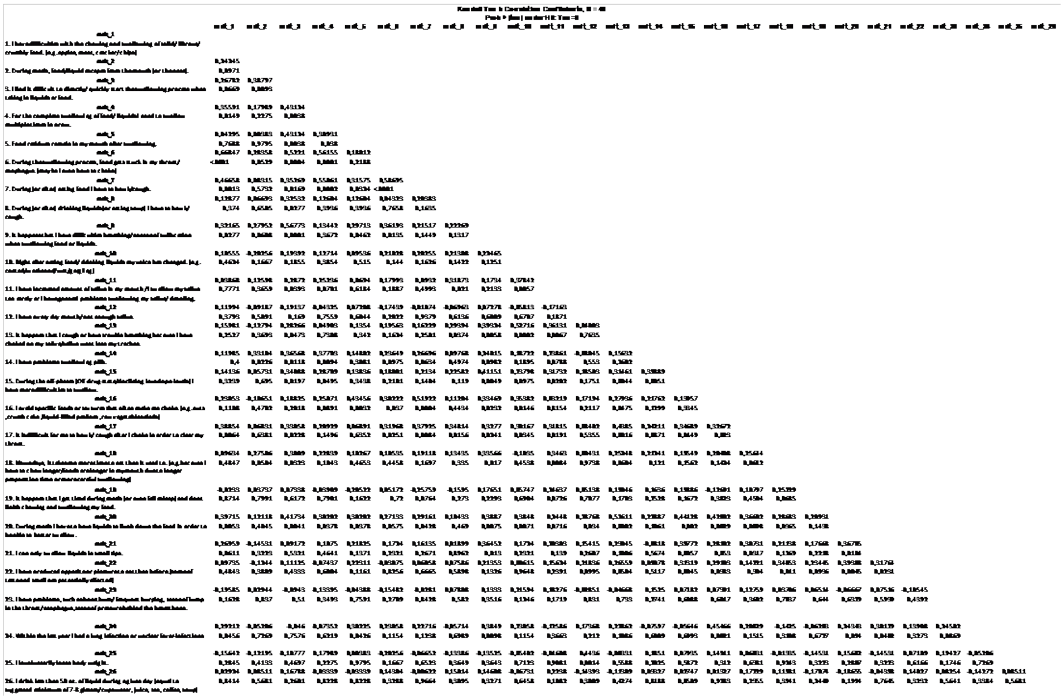

b) Correlogram French version 2 (FR2)

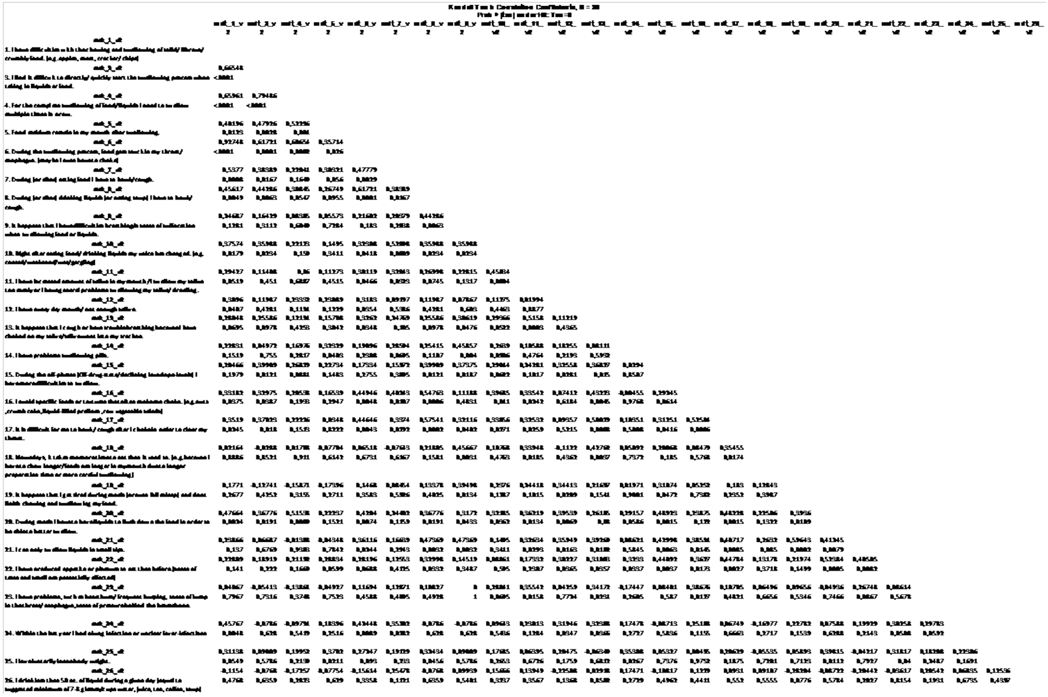

c) Correlogram German version (GE)

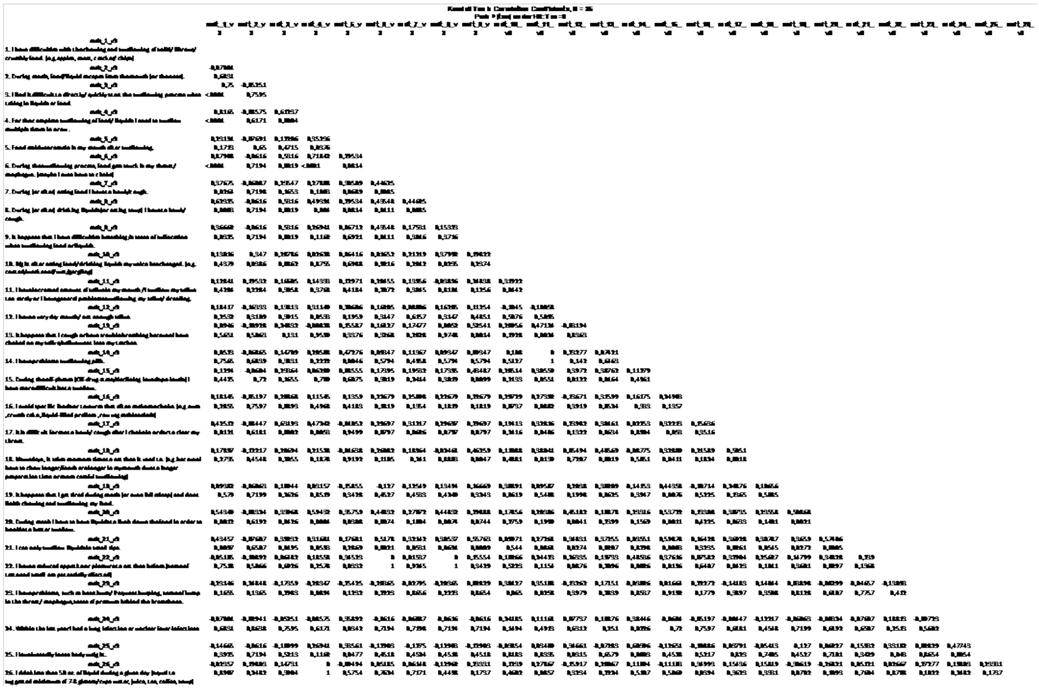

Supplement 6: Detailed analyses of MDT-PD construct validity

Table 6.1 Analysis of MDT-PD mean differences on the diagnosis of parkinsonism in FR1

Source	DF	Sum of Squares	Mean Square	F Value	Pr > F	R-Square
**Model**	5	29.8571915	5.9714383	1.07	0.3906	0.120856
**Error**	39	217.1895257	5.5689622			
**Corrected Total**	44	247.0467172				
**Diagnosis**	5	29.85719153	5.97143831	1.07	0.3906	

Table 6.2 Analysis of MDT-PD mean differences on the diagnosis of parkinsonism in FR2

Source	DF	Sum of Squares	Mean Square	F Value	Pr > F	R-Square
**Model**	4	31.3634172	7.8408543	1.56	0.2078	0.159092
**Error**	33	165.7772342	5.0235526			
**Corrected Total**	37	197.1406514				
**Diagnosis**	4	31.36341719	7.84085430	1.56	0.2078	

Table 6.3 Analysis of MDT-PD mean differences on the diagnosis of parkinsonism in GE

Source	DF	Sum of Squares	Mean Square	F Value	Pr > F	R-Square
**Model**	3	6.2593111	2.0864370	0.46	0.7133	0.043837
**Error**	30	136.5258107	4.5508604			
**Corrected Total**	33	142.7851218				
**Diagnosis**	3	6.25931110	2.08643703	0.46	0.7133	

Figure 6.1 Distribution of MDT-PD scores on the diagnosis of parkinsonism in FR1, FR2 and GE

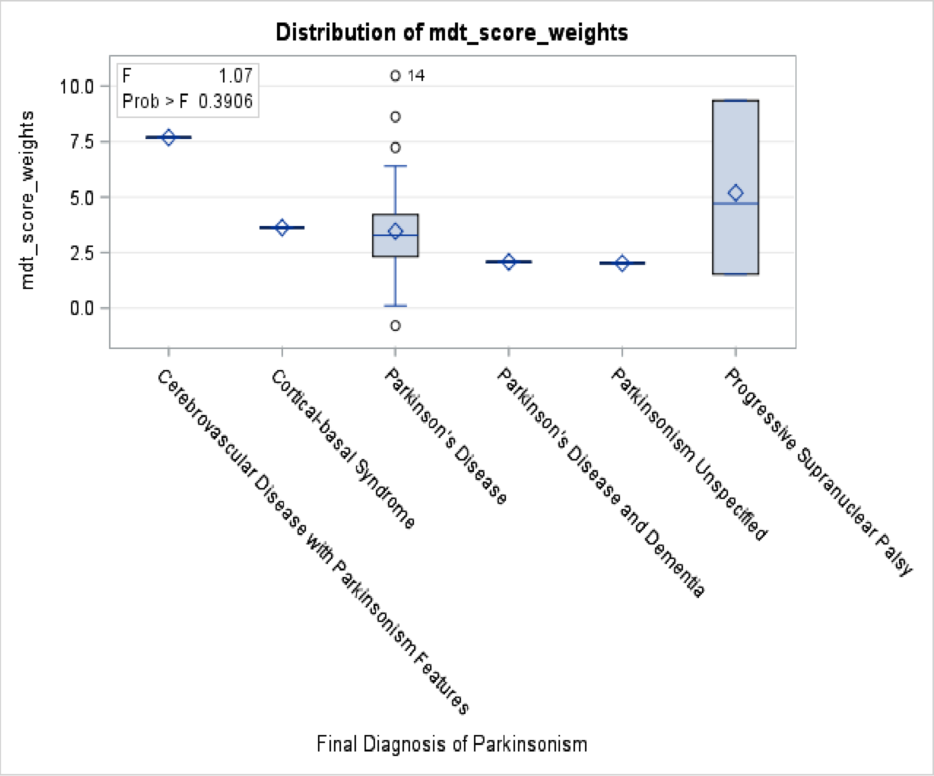

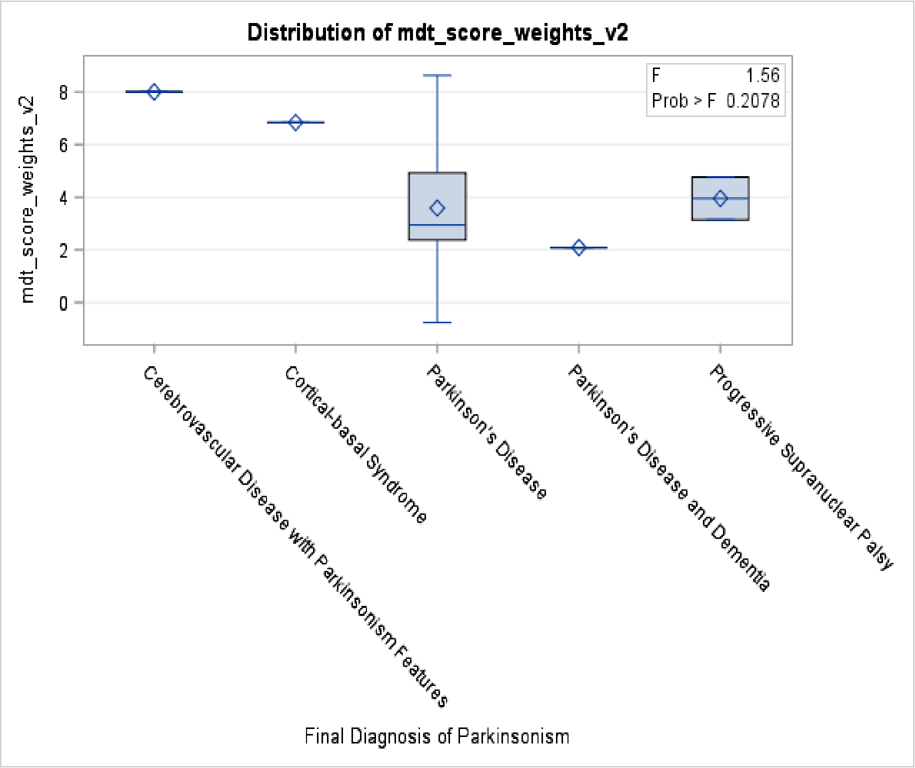

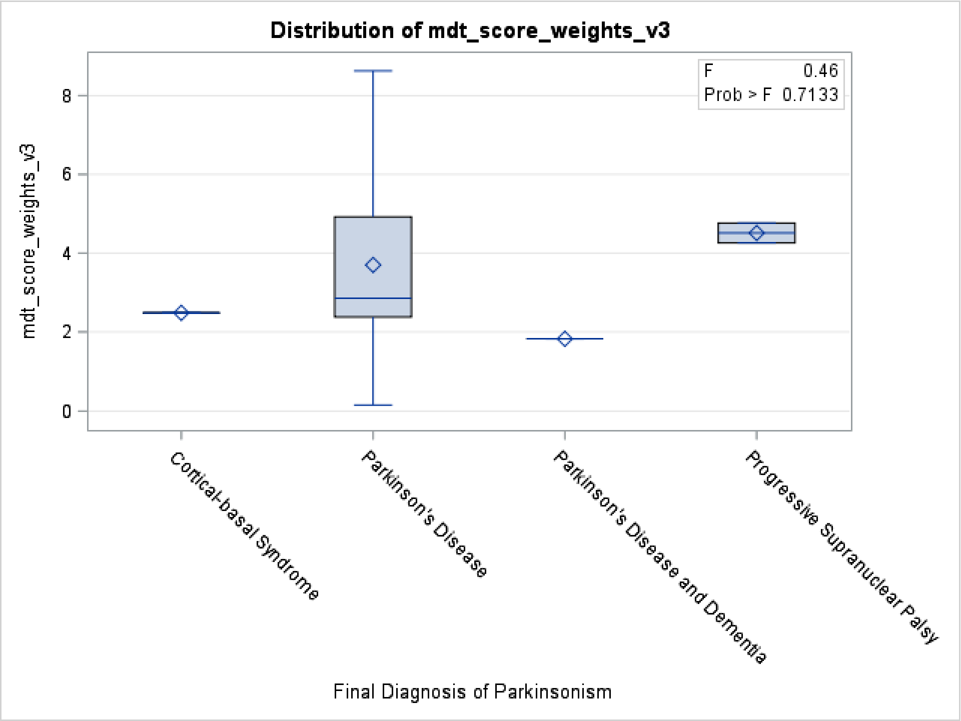

Table 6.4 Analysis of MDT-PD mean differences on Hoehn & Yahr stages in FR1

Source	DF	Sum of Squares	Mean Square	F Value	Pr > F	R-Square
**Model**	6	20.7734144	3.4622357	0.59	0.7385	0.084825
**Error**	38	224.1225919	5.8979629			
**Corrected Total**	44	244.8960063				
**H&Y**	6	20.77341438	3.46223573	0.59	0.7385	

Table 6.5 Analysis of MDT-PD mean differences on Hoehn & Yahr stages in FR2

Source	DF	Sum of Squares	Mean Square	F Value	Pr > F	R-Square
**Model**	6	16.5468600	2.7578100	0.49	0.8124	0.083814
**Error**	32	180.8771396	5.6524106			

**Corrected Total**	38	197.4239996				
**H&Y**	6	16.54685996	2.75780999	0.49	0.8124	

Table 6.6 Analysis of MDT-PD mean differences on Hoehn & Yahr stages in GE

Source	DF	Sum of Squares	Mean Square	F Value	Pr > F	R-Square
**Model**	6	3.8264889	0.6377482	0.13	0.9918	0.026712
**Error**	28	139.4256358	4.9794870			
**Corrected Total**	34	143.2521247				
**H&Y**	6	3.82648890	0.63774815	0.13	0.9918	

Figure 6.2 Distribution of MDT-scores on Hoehn & Yahr stages in FR1, FR2 and GE

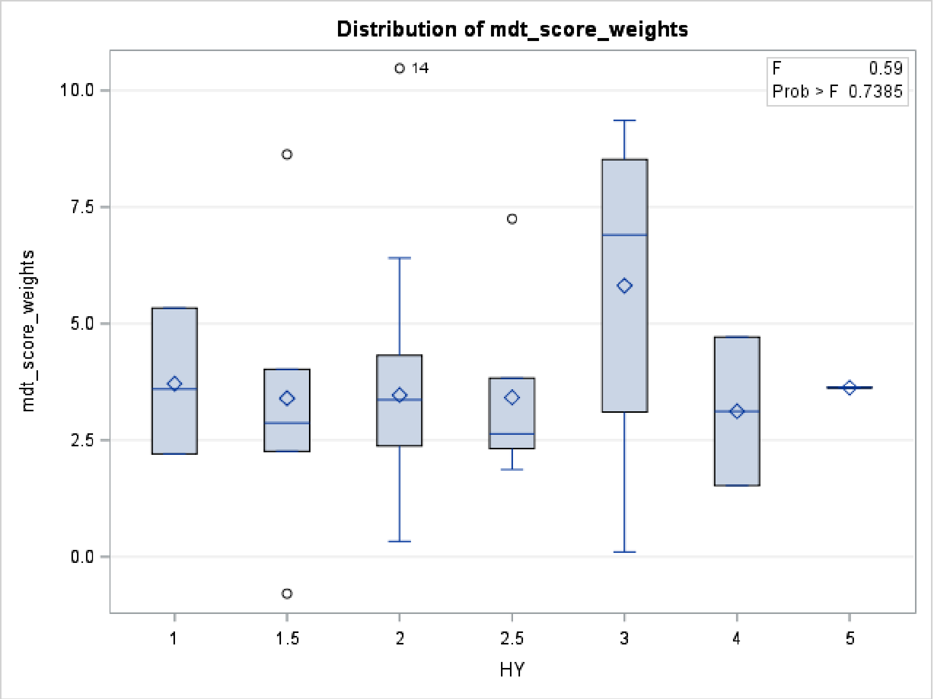

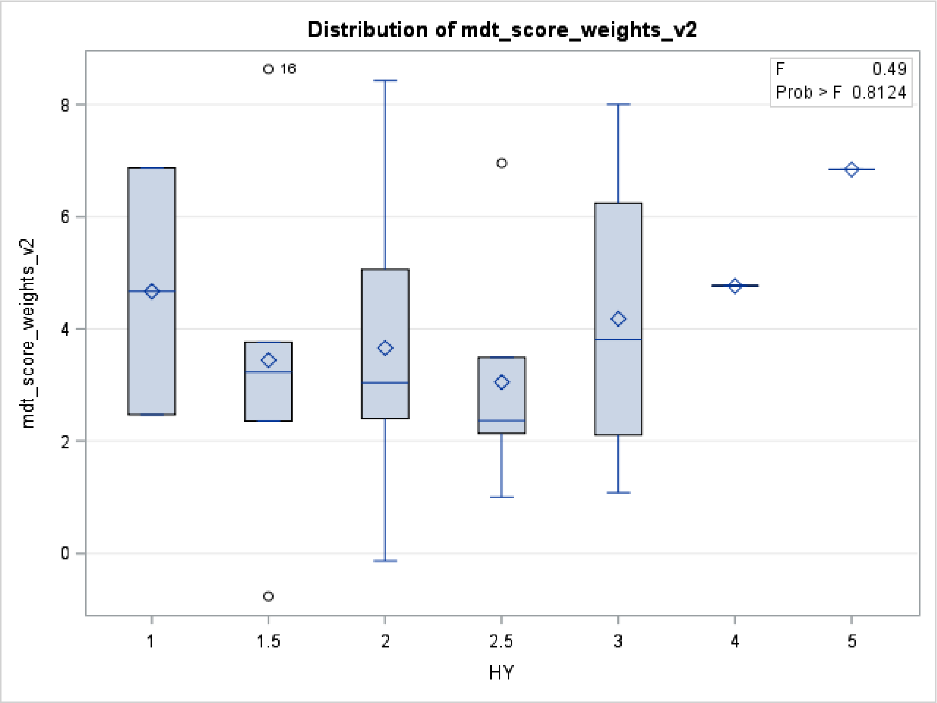
Distribution of mdt_score_weights_v2
F 0.49
Prob > F 0.8124
16
mdt_score_weights_v2
8
6
4
2
0
1
1.5
2
2.5
3
4
5
HY

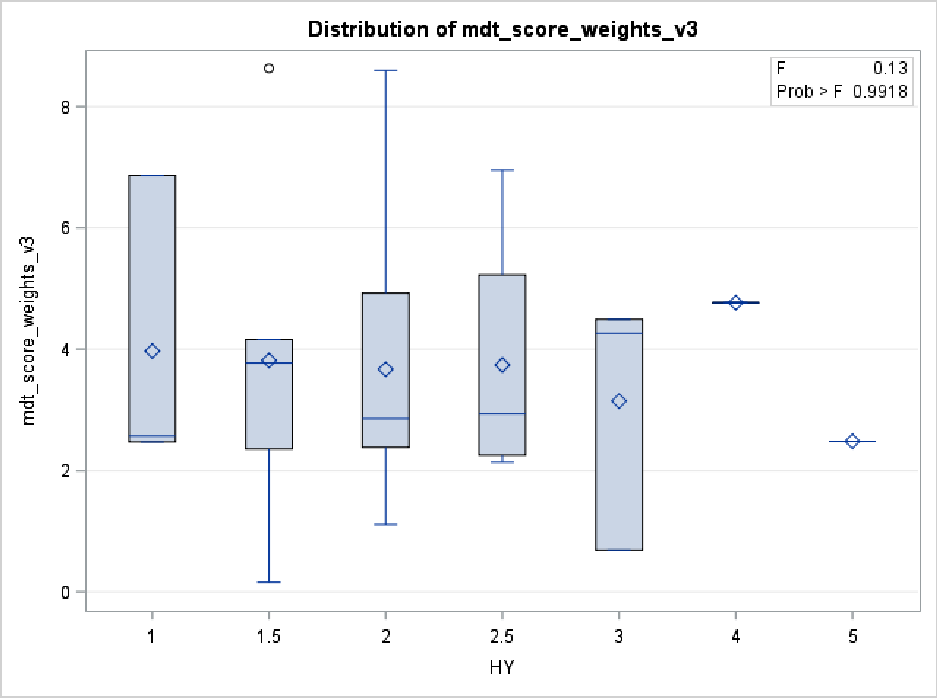
Distribution of mdt_score_weights_v3
F 0.13
Prob > F 0.9918
mdt_score_weights_v3
8
6
4
2
0
1
1.5
2
2.5
3
4
5
HY

Table 6.7 Correlation between MDT-PD and Moca scores in FR1

Source	DF	Sum of Squares	Mean Square	F Value	Pr > F
**Model**	1	4.2891936	4.2891936	0.77	0.3862
**Error**	43	240.6068127	5.5955073		
**Corrected Total**	44	244.8960063			

Table 6.8 Correlation between MDT-PD and Moca scores in FR2

Source	DF	Sum of Squares	Mean Square	F Value	Pr > F
**Model**	1	4.1356935	4.1356935	0.79	0.3793
**Error**	37	193.2883061	5.2240083		
**Corrected Total**	38	197.4239996			

Table 6.9 Correlation between MDT-PD and Moca scores in GE

Source	DF	Sum of Squares	Mean Square	F Value	Pr > F
**Model**	1	4.5272313	4.5272313	1.08	0.3069
**Error**	33	138.7248934	4.2037846		
**Corrected Total**	34	143.2521247			

Figure 6.3 Correlation between MDT-PD and MoCA scores in FR1, FR2 and GE

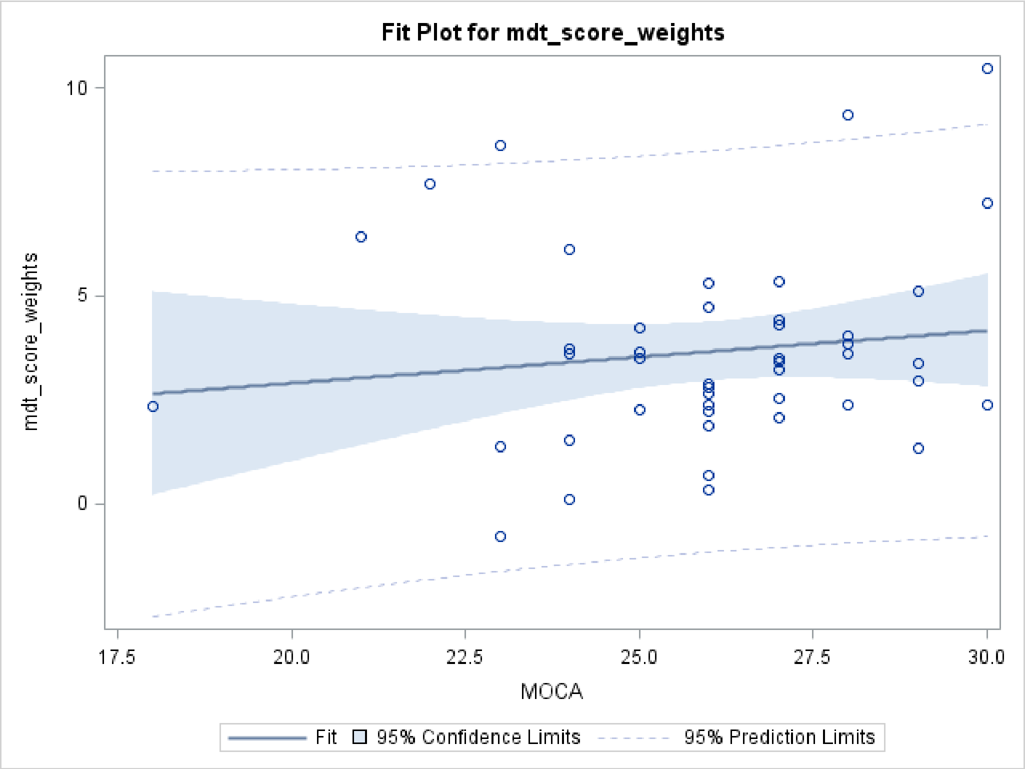

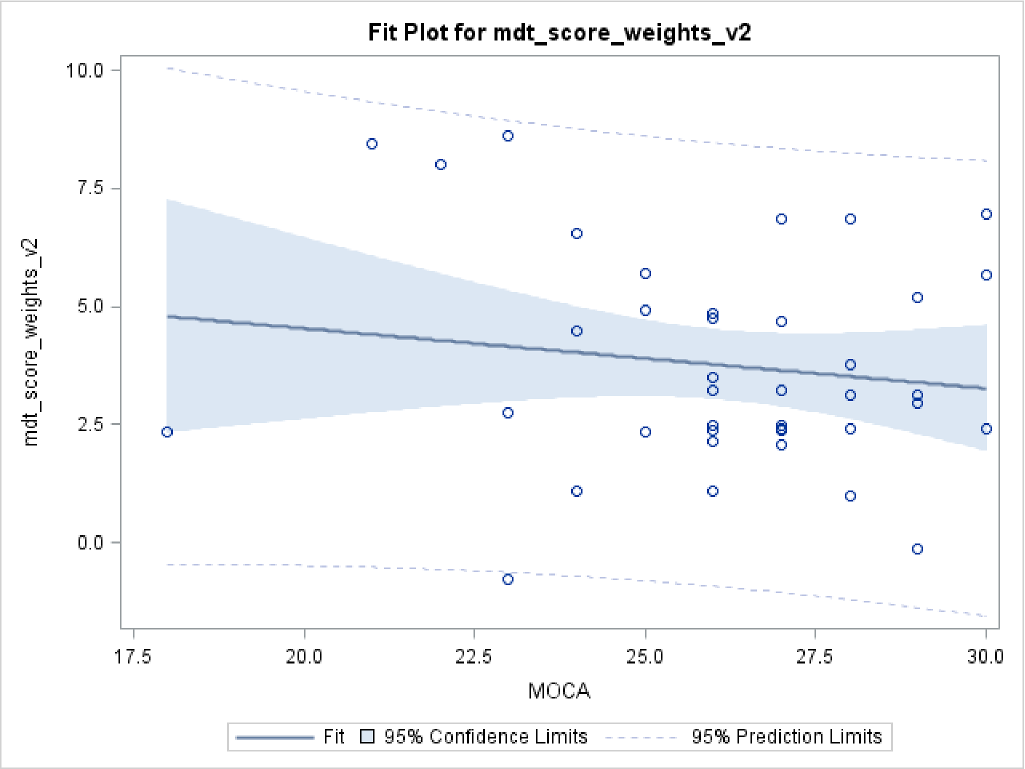

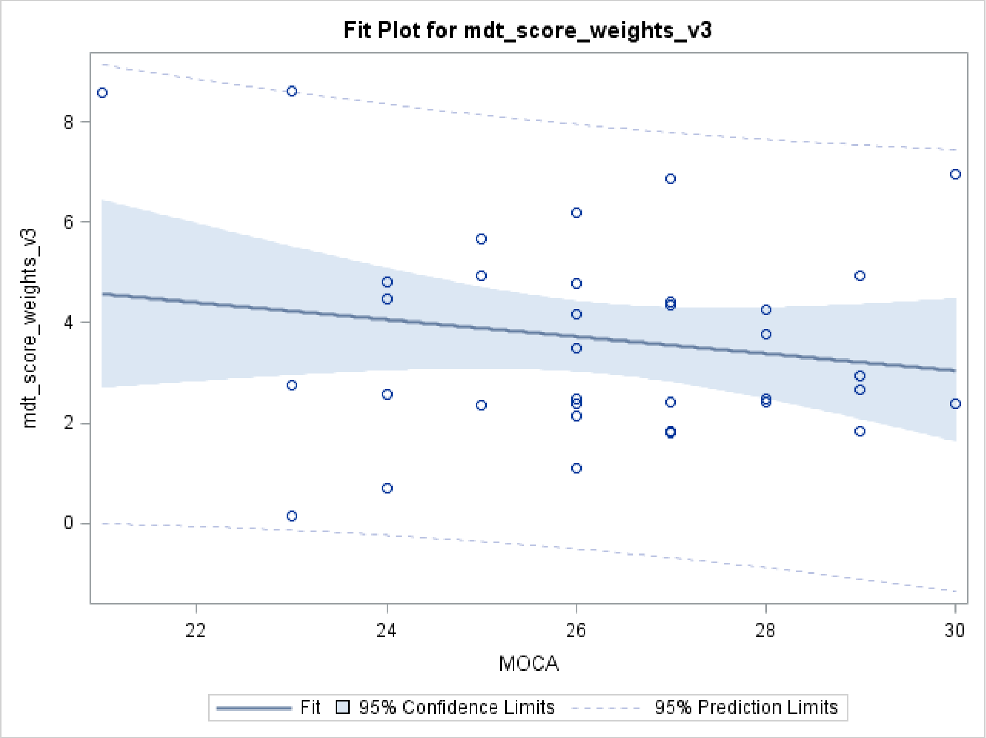

Table 6.10 Correlation between MDT-PD and MDS-UPDRSIII scores in FR1

Source	DF	Sum of Squares	Mean Square	F Value	Pr > F
**Model**	1	1.7100217	1.7100217	0.29	0.5926
**Error**	41	241.1020823	5.8805386		
**Corrected Total**	42	242.8121040			

Table 6.11 Correlation between MDT-PD and MDS-UPDRSIII scores in FR2

Source	DF	Sum of Squares	Mean Square	F Value	Pr > F
**Model**	1	0.1376729	0.1376729	0.02	0.8760
**Error**	35	195.0970618	5.5742018		
**Corrected Total**	36	195.2347348			

Table 6.12 Correlation between MDT-PD and MDS-UPDRSIII scores in GE

Source	DF	Sum of Squares	Mean Square	F Value	Pr > F
**Model**	1	1.9974334	1.9974334	0.45	0.5096
**Error**	31	139.1111016	4.4874549		
**Corrected Total**	32	141.1085350			

Figure 6.4 Correlation between MDT-PD and MDS-UPDRSIII in FR1, FR2 and GE

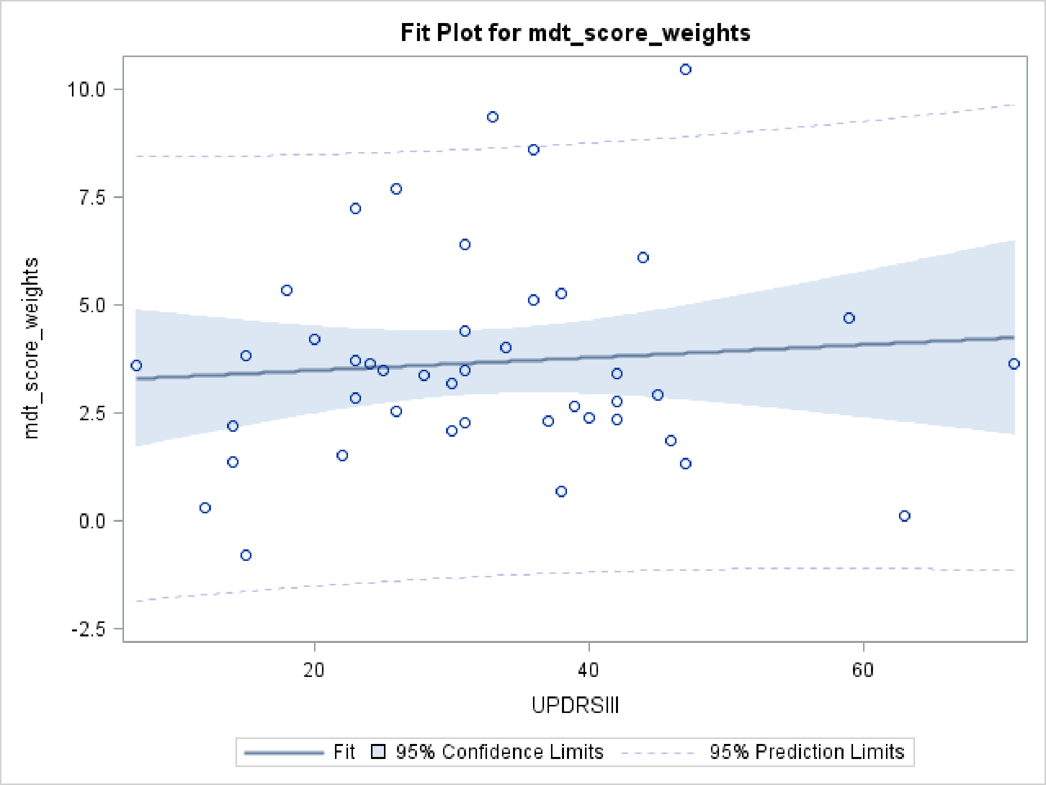

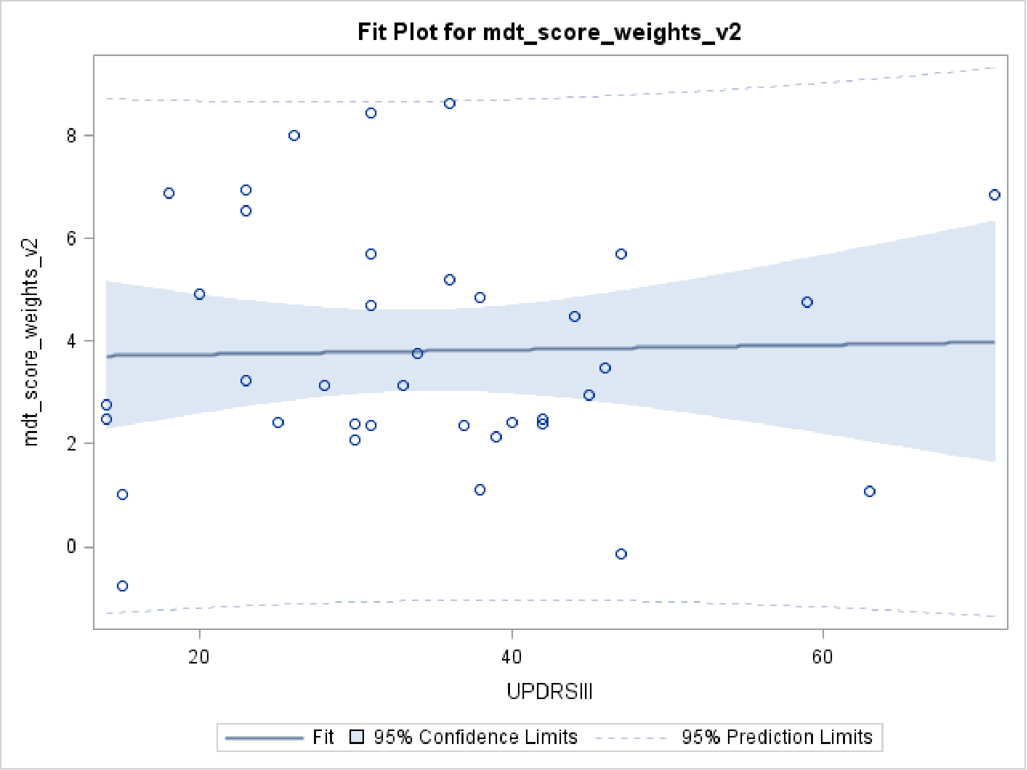
Fit Plot for mdt_score_weights_v2
mdt_score_weights_v2
UPDRSIII
0
2
4
6
8
20
40
60
Fit
95% Confidence Limits
95% Prediction Limits

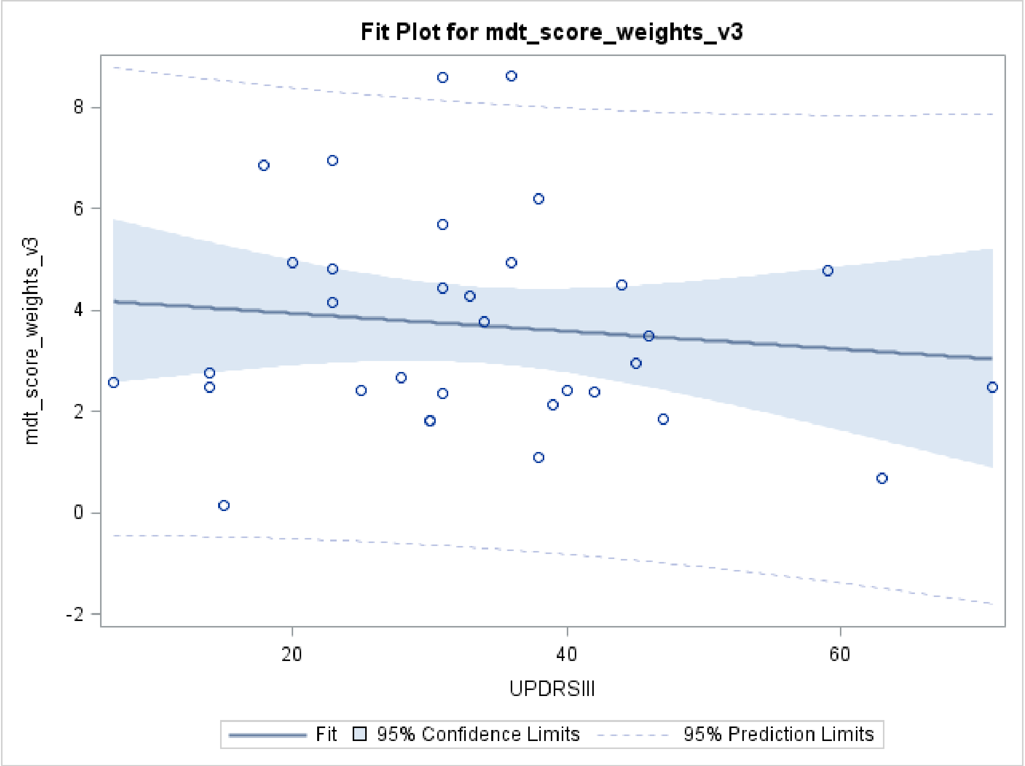
Fit Plot for mdt_score_weights_v3
mdt_score_weights_v3
UPDRSIII
-2
0
2
4
6
8
20
40
60
Fit
95% Confidence Limits
95% Prediction Limits

Table 6.13 Correlation between MDT-PD and disease duration in FR1

Source	DF	Sum of Squares	Mean Square	F Value	Pr > F	R-Square
**Model**	1	5.6354609	5.6354609	1.02	0.3179	0.022168
**Error**	45	248.5797761	5.5239950			
**Corrected Total**	46	254.2152370				

Table 6.14 Correlation between MDT-PD and disease duration in FR2

Source	DF	Sum of Squares	Mean Square	F Value	Pr > F	R-Square
**Model**	1	0.0387460	0.0387460	0.01	0.9311	0.000189
**Error**	40	204.9740164	5.1243504			
**Corrected Total**	41	205.0127624				

Table 6.15 Correlation between MDT-PD and disease duration in GE

Source	DF	Sum of Squares	Mean Square	F Value	Pr > F	R-Square
**Model**	1	0.6993853	0.6993853	0.16	0.6937	0.004239
**Error**	37	164.3002127	4.4405463			
**Corrected Total**	38	164.9995980				

Figure 6.5 Correlation between MDT-PD and disease duration in FR1, FR2 and GE and third evaluations.

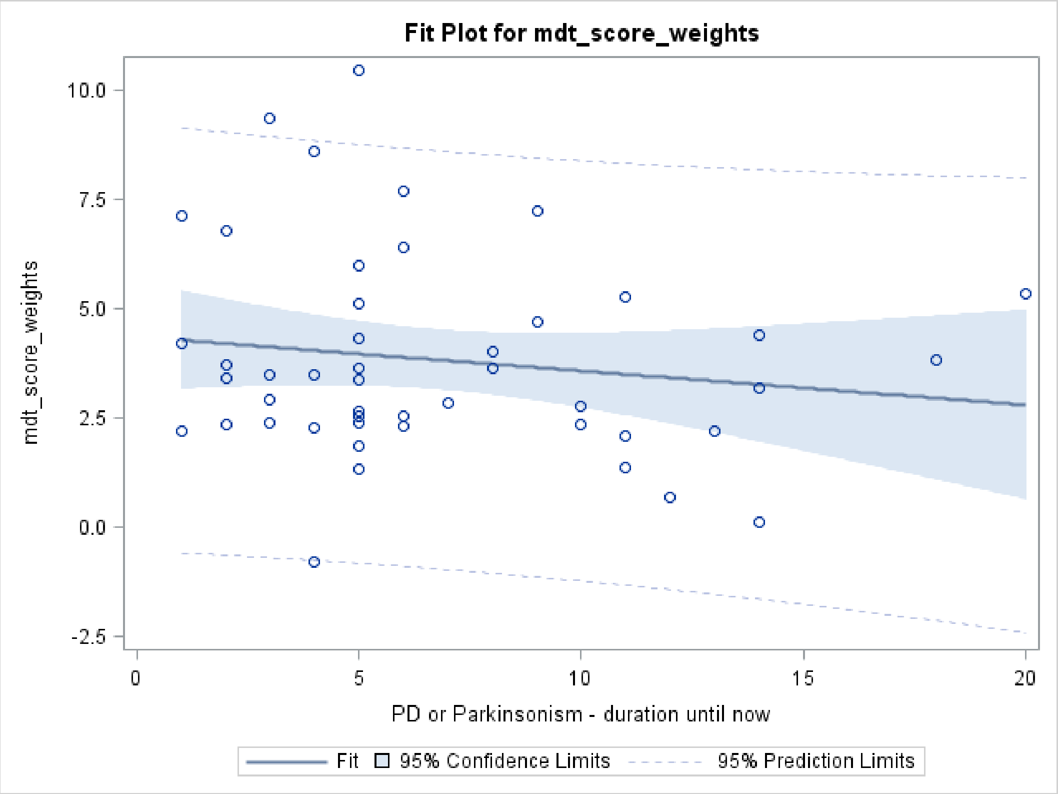

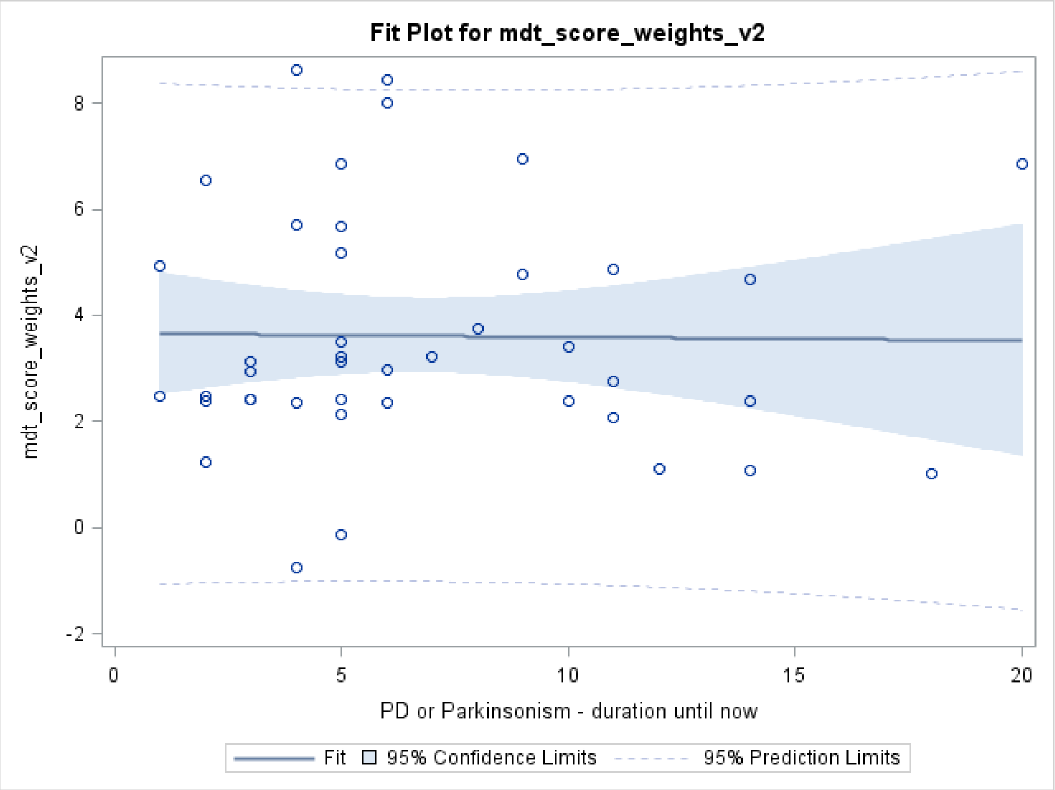

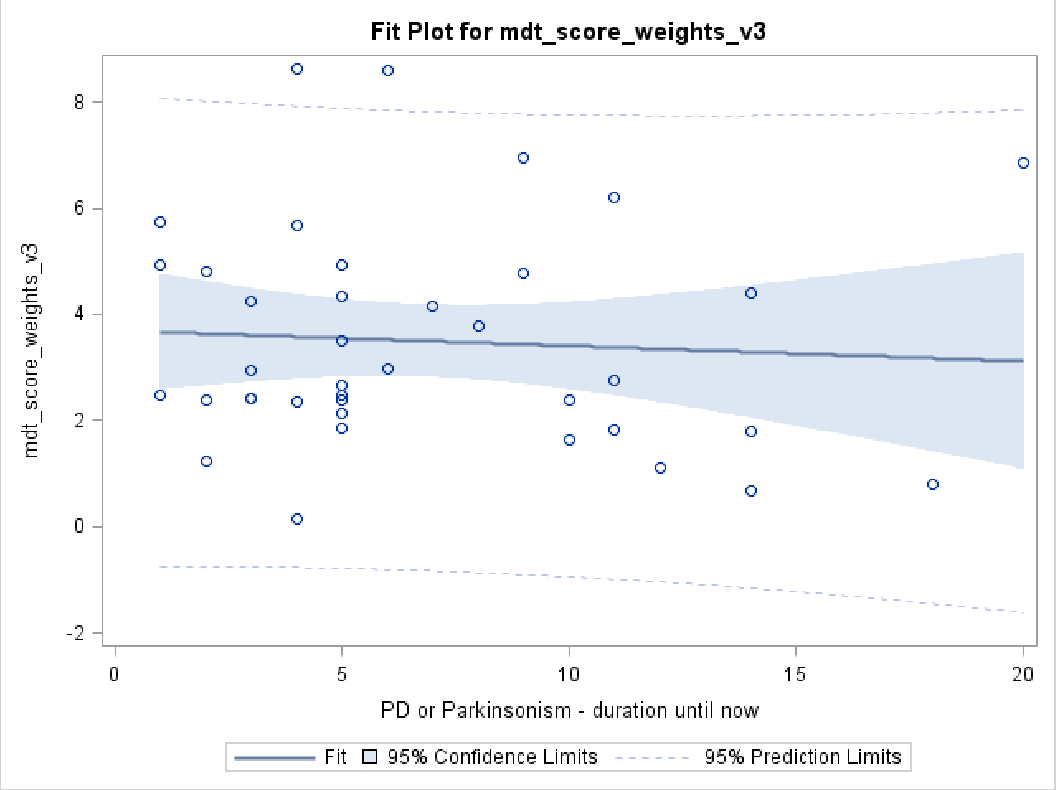
Fit Plot for mdt_score_weights_v3
mdt_score_weights_v3
8
6
4
2
0
-2
0
5
10
15
20
PD or Parkinsonism - duration until now
Fit
95% Confidence Limits
95% Prediction Limits

Supplement 7 Supplementary evaluation of MDT-PD score characteristics

Table 7.2 Distribution of the MDT-PD questionnaire score categories

	FR1	FR2	GE
**No noticeable dysphagia**	29 (63.04)	23 (58.97)	19 (54.29)
**Noticeable, oropharyngeal dysphagia**	7 (15.22)	4 (10.26)	7 (20.00)
**Dysphagia with risk of aspiration**	10 (21.74)	12 (30.77)	9 (25.71)
Total	46	39	39

Table 7.3 Descriptive statistics of the MDT-PD questionnaire score

		Weighted MDT-PD sum score		
		**FR1**	**FR2**	**GE**
**N**	valid	46	39	35
	missing	0	7	11
**Standard error of the mean**		0.35	0.36	0.35
**Mean**		3.63	3.75	3.68
**Median**		3.39	3.14	2.94
**Standard deviation**		2.35	2.28	2.05
**Skewness**		0.98	0.44	0.77
**Standard error of the skewness**		0.35	0.38	0.40
**Kurtosis**		1.23	-0.28	0.29
**Standard error of the kurtosis**		0.69	0.74	0.78
**Range**		11.26	9.39	8.47
**Minimum**		-0.79	-0.76	0.16
**Maximum**		10.47	8.63	8.63
**Percentile**	25	2.27	2.38	2.38
	75	4.42	8.63	4.81

Supplement 8 Further details for MDT-PD diagnostic discrimination power

Table 8 Comparison between differently defined severity codes for dysphagia classification in order to test the diagnostic accuracy of the MDT-PD questionnaire

Diagnostic parameter	Dysphagia severity classification		
**PAS-H_2O**	**0**	**1**	**2**
**Simons et al. 2014**	No dysphagia<br>(PAS = 1)<br>Material does not enter the airway	Oropharyngeal dysphagia<br>(PAS = 2)	Dysphagia with risk of aspiration<br>(PAS >= 3)<br>Including penetration and aspiration<br>(starting with level 3: "Material enters the airway, remains above the vocal folds, and is not ejected from the airway" until level 8: "Material enters the airway, passes below the vocal folds, and no effort is made to eject)
**Buhmann et al. 2018**	Without severe pathological findings<br>(PAS 1-2)<br>Including penetration of laryngeal structures above the vocal folds<br>(level 2: "Material enters the airway, remains above the vocal folds, and is ejected from the airway")	Penetration<br>(PAS 3-5)	Aspiration<br>(PAS 6-8)

PAS-H2O: Penetration-aspiration scale (PAS) for the consistency water (H_2O) was used together with flexible endoscopic evaluation of swallowing (FEES).

Background information about diagnostic study approaches:
For validation purpose of the MDT-PD a set of 18 diagnostic parameter were used for subjects' final dysphagia severity classification in the original study, Simons et al. 2014 (4 parameter via clinical swallowing assessment CSA, 14 parameter via FEES);
Buhmann et al. 2018 used PAS-H_2O as the only diagnostic parameter against MDT-PD (with divergent severity codes).

PAS definition after Rosenbek, JC, Robbins, J, Roecker EV, Coyle, JL, & Woods, JL. A Penetration-Aspiration Scale. Dysphagia 11:93-98, 1996
